# A cross-cultural comparison of intrinsic and extrinsic motivational drives for learning

**DOI:** 10.3758/s13415-024-01228-2

**Published:** 2024-10-18

**Authors:** Zhaoqi Zhang, Lieke L. F. van Lieshout, Olympia Colizoli, Haoqian Li, Tongxi Yang, Chao Liu, Shaozheng Qin, Harold Bekkering

**Affiliations:** 1 State Key Laboratory of Cognitive Neuroscience and Learning, IDG/McGovern Institute for Brain Research, Beijing Normal University, Beijing, China; 2 Donders Institute for Brain, Cognition and Behaviour, Radboud University, Nijmegen, Netherlands; 3 Chinese Institute for Brain Research, Beijing, China

**Keywords:** Motivation, Cross-cultural comparison, Autonomy, Reward, Learning

## Abstract

Intrinsic motivational drives, like the autonomous feeling of control, and extrinsic motivational drives, like monetary reward, can benefit learning. Extensive research has focused on neurobiological and psychological factors that affect these drives, but our understanding of the sociocultural factors is limited. Here, we compared the effects of autonomy and rewards on episodic recognition memory between students from Dutch and Chinese universities. In an exploratory learning task, participants viewed partially obscured objects that they needed to subsequently remember. We independently manipulated autonomy, as volitional control over an exploration trajectory, as well as the chance to receive monetary rewards. The learning task was followed by memory tests for objects and locations. For both cultural groups, we found that participants learned better in autonomous than non-autonomous conditions. However, the beneficial effect of reward on memory performance was stronger for Chinese than for Dutch participants. By incorporating the sociocultural brain perspective, we discuss how differences in norms and values between Eastern and Western cultures can be integrated with the neurocognitive framework about dorsal lateral and ventral medial prefrontal cortex and dopaminergic reward modulations on learning and memory. These findings have important implications for understanding the neurocognitive mechanisms in which both autonomy and extrinsic rewards are commonly used to motivate students in the realm of education and urge more attention to investigate cultural differences in learning.

**Supplementary Information:**

The online version contains supplementary material available at 10.3758/s13415-024-01228-2.

## Introduction

Learning is a crucial aspect of life: it is the ability to acquire knowledge and skills that are essential for personal and professional development. Motivation is the driving force that initiates and sustains learning efforts (Murayama & Jach, 2024; Ryan & Deci, 2017). Considerable research has concentrated on exploring only the biological and psychological aspects influencing motivation for learning (e.g., Di Domenico & Ryan, 2017), not on the equally crucial sociocultural factors, even though cultural backgrounds shape both behavior and brain development (neuroplasticity) by changing values, beliefs, expectations, and cognitive processes (Han et al., 2013; Kitayama & Salvador, 2024; Park & Huang, 2010; Qu et al., 2021). In the current study, we aim to fill this research gap by investigating how diverse cultural backgrounds, taking Chinese and Dutch cultures as examples, interact with the beneficial effects of intrinsic and extrinsic motivation on learning.

One of the key theories about motivation, Self-Determination Theory (SDT), proposed seeing motivation as a continuum ranging from extrinsic motivation to intrinsic motivation (Ryan & Deci, 2000; Ryan & Deci, 2020). Extrinsic motivation comes from external sources (e.g., monetary reward) and can improve learning performance (Adcock et al., 2006; Duan et al., 2020; Elliott et al., 2020; Mason et al., 2017; Murayama & Kuhbandner, 2011). Intrinsic motivation, in contrast, refers to the internal desire and enjoyment derived from engaging in an activity (Ryan & Deci, 2000), and can also enhance learning performance (Duan et al., 2020; Gruber et al., 2014; Gruber & Ranganath, 2019; Jepma et al., 2012; Kang et al., 2009; Ripolles et al., 2016). Intrinsic motivation can be fostered by satisfying our basic psychological needs (i.e., the need for autonomy, competence, and relatedness; Deci & Ryan, 1985). Among these needs, autonomy, referred to as self-controllable to choose, stands out as a particularly critical element, since autonomy not only supports but also initiates behaviors (Leotti et al., 2010). Fulfilling the need of autonomy helps with learning and memory (Bramley et al., 2016; DuBrow et al., 2019; Izuma et al., 2010; Kaplan et al., 2012; Markant et al., 2014; Markant & Gureckis, 2014; Murty et al., 2015; Rotem-Turchinski et al., 2019; Voss & Cohen, 2017; Voss, Galvan et al., 2011a, Voss, Gonsalves et al., 2011b, Voss, Warren et al., 2011c). In learning experiments, autonomy can be fostered by giving participants the choice of which button to press (Ding et al., 2021; DuBrow et al., 2019; Murty et al., 2015) or by allowing them to freely control their learning trajectory (Kaplan et al., 2012; Markant et al., 2014; Voss, Galvan et al., 2011a, Voss, Gonsalves et al., 2011b, Voss, Warren et al., 2011c).

Although SDT posits that motivation can be categorized into intrinsic and extrinsic types, human functional neuroimaging research has revealed that the underlying mechanisms of both intrinsic and extrinsic motivation exhibit both dissociation and overlap. Reward-motivated learning could elicit functional activation and connectivity among a network of distributed regions, including the orbital (OFC) and ventral medial prefrontal cortex (VMPFC) and dopaminergic circuitry, including the ventral tegmental area (VTA), midbrain, and ventral striatum (Adcock et al., 2006; Sescousse et al., 2013; Shigemune et al., 2014; Wolosin et al., 2012). Autonomy-motivated learning, in contrast, not only elicits activation and connectivity of the abovementioned brain regions but also engages the higher-order prefrontal network including the dorsal lateral prefrontal cortex (DLPFC; Murty et al., 2015; Voss, Galvan et al., 2011a, Voss, Gonsalves et al., 2011b, Voss, Warren et al., 2011c). These findings suggest a complex interplay where motivational types are not entirely distinct but share common neural substrates. While there is considerable evidence investigating the mechanism of extrinsic and intrinsic motivation in learning, discourse on how cultural factors shape these motivational factors remains inconclusive, as these studies yielded diverse results.

There has been abundant evidence suggesting that cultural backgrounds can alter how people perceive extrinsic motivators, for example, monetary rewards. This was mostly discussed under the premise of working environments. For instance, Chinese employees would become more devoted to their tasks when their monetary income increased, while for American employees, their devotion to their jobs was not relevant to their income (Huang, 2013). Similarly, Tang et al. (2003) also found that Chinese employees had higher respect for money compared to American and British employees. Furthermore, it has been observed that individuals who identify themselves more closely with collectivistic cultures tend to be extrinsically motivated to achieve their career goals (Arshad et al., 2019). This finding was also validated by ample educational studies investigating differences in motivation for learning between Eastern and Western cultures. In Eastern educational contexts, factors that come from external environments are more emphasized than in Western educational contexts, like materialistic rewards, academic achievement, expectancy of success, and group benefits (Blevins et al., 2023; Chen et al., 2005; Iyengar & DeVoe, 2003; Telzer et al., 2017). This could result in students from the East exhibiting anxiety about their learning performance and achievement motivation (Essau et al., 2008). In contrast, the anxiety of students from Germany was found to not be correlated with learning performance. Years of emphasis on these different forms of external drives might lead to a stronger adoption of extrinsic motivation for students from an Eastern culture. For instance, it was found that extrinsic motivation contributed to the achievement level in mathematics in Eastern students whereas it even had a detrimental effect on the achievement level in mathematics in Western students (Zhu & Leung, 2011). A neuroimaging study demonstrated that the activation and connectivity between the inferior frontal gyrus and the ventral striatum (part of the dopaminergic circuitry) exhibit greater stability and persistence among Asian students compared to American students. This was observed in response to a boring go/no-go task where Asian and American participants were asked to improve their performance. In the American group, this neural coupling and activation tended to decrease over time (Telzer et al., 2017). This was also in line with the neuroplastic theory of culture-brain interaction. Specifically, the cultural environment might have an impact on top-down modulation of subcortical regions (e.g., dopaminergic circuitry) during emotional or motivational processes (Chiao, 2015).

However, recent studies have indicated that in some situations Western participants might be more sensitive to rewards than Eastern participants (Liu et al., 2020; Medvedev et al., 2024). For example, Medvedev et al. (2024) found that the drive for monetary rewards on task performance was stronger for participants from Western countries than those from Eastern countries. Furthermore, it was also found by one neuroimaging study that reward circuitry activation did not differ between cultural groups when participants received monetary rewards (Blevins et al., 2023). Therefore, the consensus on how extrinsic motivation influences behaviors across cultures is not uniform, prompting further exploration into this complex topic.

Similarly, evidence regarding cross-cultural differences in intrinsic motivation for learning presents a varied perspective. Some studies have suggested that personal choices are more valuable for students from Western cultures than for students from Eastern cultures (Iyengar & Lepper, 1999; Markus & Kitayama, 2003; Sastry & Ross, 1998). This could be explained by potential differences in the origins of intrinsic motivation to learn between Eastern and Western cultures (Liu et al., 2020). They elaborated that for European students, intrinsic motivation usually comes from their own interest in learning (i.e., autonomy). However, for Eastern students who were deeply influenced by Confucian philosophy, their intrinsic learning motivation comes from the internalization of the importance of learning. In other words, they derived a strong personal belief that learning is important for their future development, social status, and career success, despite their lack of interest in the learning content. These differences in values also might shift learning styles and preferences. For example, Chinese students embrace teacher-led instruction, aligning with cultural norms of respect for guidance, whereas American students often view the same approach as constraining and prefer a more self-dependent learning style (Zhou et al., 2012).

Alternatively, there is sufficient evidence suggesting that the beneficial effect of autonomy for learning is universal across Eastern and Western cultures (Chirkov et al., 2003; Chirkov, 2009; Chirkov et al., 2010; Helwig, 2006; Nalipay et al., 2020; Ryan & Deci, 2006; Vansteenkiste et al., 2005, 2006, 2020; Wichmann, 2011; Yu et al., 2016). Although it is more intuitive to think that autonomy is a Western philosophical concept, Eastern Confucian culture has also been emphasizing the importance of personal choices (i.e., autonomy) in learning, conceptualized as “self-cultivation” (Ryan & Deci, 2017). This was also in line with the Basic Psychological Needs Theory in SDT suggesting that autonomy is an instinctive psychological need, and it is not influenced by social contexts (Ryan & Deci, 2017; Vansteenkiste et al., 2020). In summary, further research is required to understand if there is a cultural difference in intrinsic motivation for learning between Eastern and Western cultures.

Interestingly, the interaction between extrinsic and intrinsic motivation in learning has been controversial. On one hand, several studies have suggested that extrinsic motivation can undermine intrinsic motivation for learning (Deci & Koestner, 1999; Hidi, 2015; Murayama et al., 2010; van Lieshout et al., 2023), and vice versa. For instance, Murayama and Kuhbandner (2011) found that the effect of extrinsic motivation on learning would also be undermined when students are learning interesting content. This negative interaction between intrinsic and extrinsic motivation in learning was proposed by the “over-justification hypothesis” (Lepper et al., 1973). This hypothesis states that when people are rewarded externally for their behavior, they lose interest and joy in their task (Deci & Koestner, 1999). This interaction between intrinsic and extrinsic motivation also corroborates the discovery of overlapping neural mechanisms engaged in both types of motivation (Voss, Gonsalves, et al., 2011b; Wolosin et al., 2012). In other words, intrinsic and extrinsic motivation would influence each other because they engage a similar brain mechanism. When the reward circuitry is already activated by external stimuli, the additional enhancing effect of intrinsic motivation on brain activation becomes redundant. On the other hand, there is also abundant evidence supporting the notion that intrinsic and extrinsic motivation improve learning independently. That is, people feel intrinsically engaged in learning tasks regardless of external stimulants (Duan et al., 2020). The different results in these studies may stem from an overgeneralization of the circumstances (Eisenberg, 2002). For instance, Cerasoli et al. (2014) found that rewards salient to task performances (e.g., end-of-year bonuses) could undermine intrinsic motivation, while rewards not related to task performances (e.g., basic salary) do not undermine intrinsic motivation. It was also proposed that the Eastern population might be more intrinsically motivated to work with external regulation from other people, whereas the Western population might be less intrinsically motivated to work with outside control (Eisenberg, 2002). However, there is still a research gap regarding how cultural backgrounds shape the interaction between extrinsic and intrinsic motivation within learning environments.

In the current study, we aimed to address a gap in the literature concerning how culture may interact with our motivation to learn. To do so, we investigated how intrinsic and extrinsic motivation improve learning under different cultural backgrounds, taking Chinese students and Dutch students as samples. An exploratory learning task from Voss, Galvan et al. (2011a), Voss, Gonsalves et al. (2011b), and Voss, Warren et al. (2011c) was adopted, in which participants viewed partially obscured images that they needed to subsequently remember. The learning task was followed by a separate recognition memory test. Crucially, Voss, Galvan et al. (2011a), Voss, Gonsalves et al. (2011b), and Voss, Warren et al. (2011c) found a robust main effect of autonomy on memory performance, comparing the condition when participants had control over their learning trajectory (MOVE, autonomous) with the condition in which they were asked to follow the exploratory trajectory of another participant (FOLLOW, non-autonomous). With this manipulation, we were able to control the visual information displayed as well as the movements of the joystick during the autonomous and non-autonomous conditions. In addition to the main effect of autonomy, we introduced an additional reward manipulation. Participants had the chance to receive additional monetary rewards for correctly remembering the objects during half of the exploratory learning task (extrinsic motivation; van Lieshout et al., 2023). In this way, we compared the effects of these two motivational factors (i.e., autonomy and reward) on learning between the two cultural groups of interest.

To preview, we found that extrinsic motivation (i.e., rewards) improved recognition memory for Chinese students more than for Dutch students. Furthermore, it was observed that the beneficial effect of autonomy on learning performance did not differ between Dutch and Chinese students. Lastly, based on previous literature (Liu et al., 2020), we conducted exploratory analyses by separating each cultural group into high achievers and low achievers based on their memory test performance. For Chinese students, extrinsic motivation was beneficial for both high and low achievers regardless of the existence of intrinsic motivation. In contrast, for Dutch students, extrinsic motivation did enhance learning except for high achievers when they had autonomy in learning.

In summary, investigating how intrinsic and extrinsic motivational drives affect recognition memory performance across cultures can deepen our comprehension of individual differences in how these motivational factors shape learning and behavior. This understanding can also shed light on how educational settings can be optimally improved by considering the impact of cultural background on motivation for learning. Our findings also spur debate about the neurocognitive mechanisms that underpin motivational drives and memory modulation in different cultures from the perspective of neuroplasticity and the socio-cultural brain (Han et al., 2013).

## Methods

### Preregistration and data availability

The study was preregistered on the Open Science Framework (osf.io/5bkte). All data and code used for the experimental procedure and data analyses are freely available on the Donders Repository (10.34973/tccj-j019). Part of the data on Dutch students came from the data collected by van Lieshout et al. (2023). We collected more data to match the power analysis for a between-group comparison. The experimental procedure was repeated at Beijing Normal University, Beijing, China.

### Participants

A power analysis was conducted to determine the sample size of the current study with MorePower (Campbell & Thompson, 2012). The power analysis suggested that we need at least 42 participants in each cultural group so that we can detect a medium effect size (partial *η*^*2*^ = 0.09, alpha level *p* < 0.05) with 80% power for the three-way interaction among the two within-group factors (autonomy and reward) and one between-group factor (cultural group) using a 2 × 2 × 2 mixed-measures ANOVA.

Data from 37 Dutch participants were from van Lieshout et al. (2023), among whom one participant exhibited a recognition memory test accuracy of lower than three standard deviations from the mean of the Dutch group. Additionally, we recruited ten more Dutch participants to match the power analysis, among whom one participant was excluded due to being reported as not attentive in the experiment. In the final analysis, 45 Dutch participants were included (age = 24.36 ± 5.18 years, female = 29, male = 15, non-binary = 1). Most participants were right-handed (eight left-handed, one ambidextrous). All Dutch participants had normal or corrected-to-normal vision. All Dutch participants gave written informed in line with the tenets of the Declaration of Helsinki prior to participation. The experiment was approved by the local ethics committee (CMO Arnhem-Nijmegen, The Netherlands) under a general ethics approval protocol (“Imaging Human Cognition,” CMO 2014/288) and was conducted in compliance with these guidelines. Participants were told that they would get 14 euros as standard participation compensation, while they might earn a maximum of 5 euros extra based on their task performance. All participants in the Dutch group were living, studying, or working in the Netherlands when they participated. According to official demographic information data on students at Radboud University, Nijmegen (https://www.ru.nl/en/about-us/organisation/facts-and-figures/education), we could estimate that about 90% of the Dutch participants in this dataset were local Dutch people and the other 10% comprised a majority of German students.

In Beijing, China, we recruited 55 participants, of whom we excluded 11. Seven of these excluded participants only saw less than two-thirds of the objects in one of the conditions. Three participants were excluded due to being reported as not being attentive in the experiment. We included 45 participants (age = 22.36 ± 1.92 years, female = 28, male = 17) in the final analysis for the Chinese group. All Chinese participants were right-handed and had normal or corrected-to-normal vision. All Chinese participants gave written informed consent according to the tenets of the Declaration of Helsinki prior to participation. The experiment was approved by the ethics committee of Beijing Normal University (ICBIR_A_0071_017). Participants were told that they would get 90 RMB as standard participation compensation, while they might earn a maximum of 30 RMB extra based on their task performance. Participant compensation adhered to the standard rates established by each university's regulations, with the remuneration provided in Beijing being marginally lower than that in the Netherlands. All participants in the Chinese group were local Chinese students.

During the experiment, there was a FOLLOW condition in which participants were asked to move the joystick following the searchlight trajectory shown on the screen. The trajectory in the FOLLOW condition was the recorded searchlight trajectory in the MOVE condition from the previous participant. This is the “yoking” system in the current design. Therefore, in each cultural group, the very first participant was considered a "seed” participant, (i.e., Participant 0) and this participant only did the MOVE condition. Their searchlight trajectory was shown to Participant 1, but data from Participant 0 was not included in the final analysis.

### Materials

Six hundred images were selected for visibility, recognizability, and lack of lettering from the set “2400 Unique Objects” from Brady et al. (2008). These images were presented on 24-in. full HD LED thin-film-transistor liquid-crystal display screens (1,920 × 1,080 pixels) in a square 5 × 5 grid consisting of 25 images. Experimental conditions, such as the refresh rate of the screens used for presenting stimuli, were closely matched across the test environments in China and the Netherlands. The images were 120 pixels in height and covered by black and white Gaussian noise (SD = 3). The searchlight window that uncovered the images during the learning phase was a circle with a diameter of 180 pixels. Participants could control the searchlight window with a Logitech® Attack™ 3 joystick. The experiment was programmed using PsychoPy version 3 (Peirce & MacAskill, 2018).

### Procedure

**Fig. 1:**
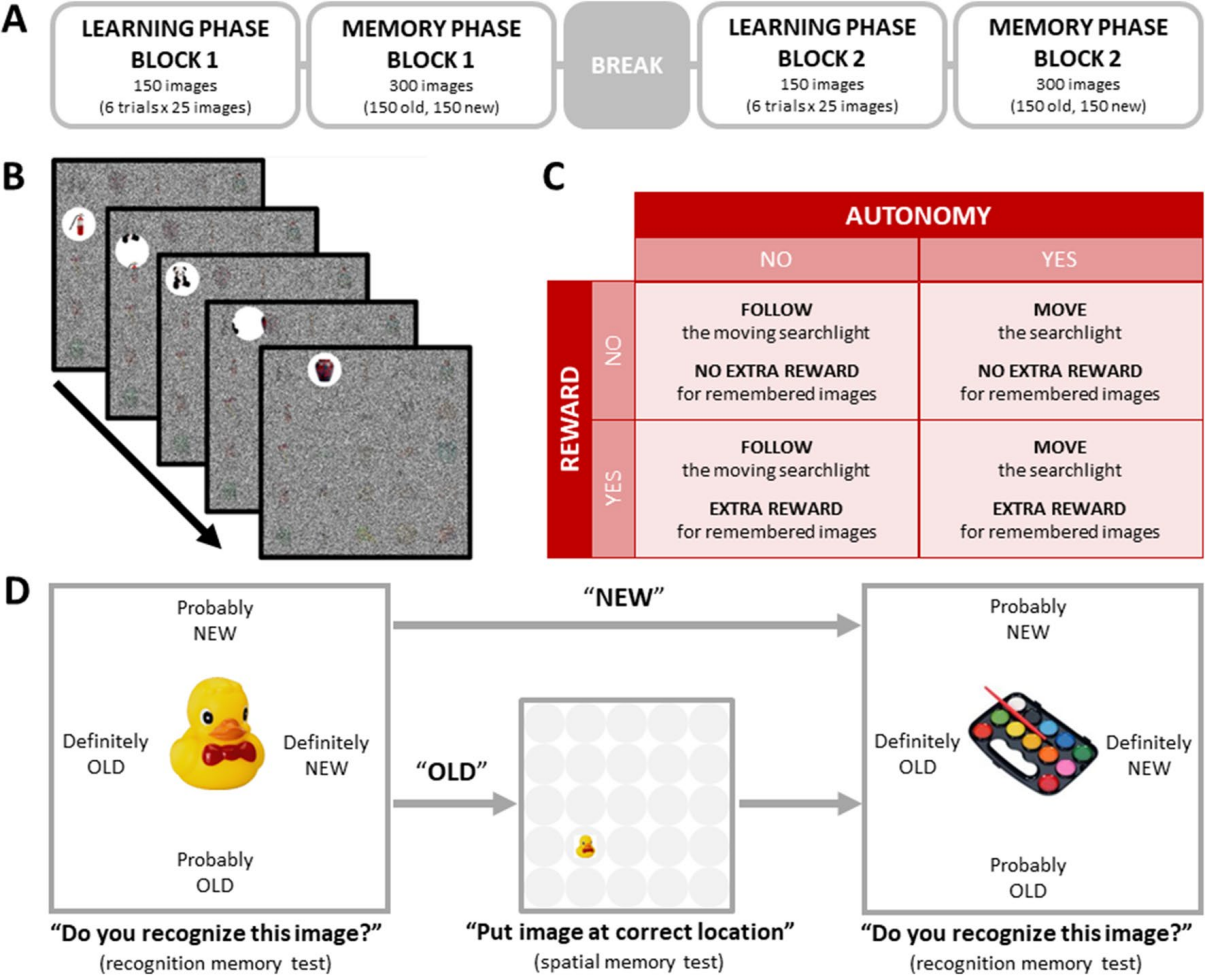
Experiment schematics. The figure is the same as Fig. 1 in van Lieshout et al. (2023). **A.** *Experimental procedure.* The whole experiment is divided into two blocks. Each block included one learning phase and one memory phase. In each learning phase, participants were shown six learning grids, and each learning grid was formed by a 5 × 5 grid containing 25 objects. After each learning phase, there would be a memory phase, in which participants would be shown 300 objects, including 150 presented objects in the last learning phase and 150 foil/new objects. **B.** *Learning grid example.* The paradigm was adapted from Voss, Galvan et al. (2011a), Voss, Gonsalves et al. (2011b), Voss, Gonsalves et al. (2011c) and previously used as described here in van Lieshout et al. (2023). In each learning grid, the 5 × 5 grid was covered by black-and-white Gaussian noise. The grid could be explored and uncovered by a moving searchlight window. Participants were told that they needed to remember as many objects as possible. **C.** *Conditions in the learning phase.* In MOVE (autonomous learning) grids, participants were instructed to control the searchlight window by moving the joystick. In FOLLOW (non-autonomous learning) grids, participants were told that the searchlight would move by itself, and they needed to use the joystick to follow the trajectory of the searchlight. Note that the trajectory of the searchlight in a FOLLOW grid was a MOVE grid trajectory recorded from the previous participant (according to a commonly used procedure called “yoking”). A learning grid might be REWARDED, in which participants would earn extra monetary rewards for recognizing the objects in that grid in the memory phase. If a learning grid was a NO REWARD grid, participants would not earn extra money for remembering these objects. Before each learning grid, participants would be shown an instruction screen, on which participants would be informed whether the following learning grid will be MOVE or FOLLOW and REWARD or NO REWARD. **D.** *Memory trial example.* Following the learning phase, there would be a memory phase in each block. In each memory phase trial, participants were asked to indicate whether the object was “Definitely OLD,” “Probably OLD,” “Probably NEW,” or “Definitely NEW.” During this recognition memory test, four reactions were located in four directions of the object, and participants could react by moving the joystick in the corresponding direction. If participants reacted such that the current object is “Definitely OLD” or “Probably OLD,” a spatial memory test would be generated for this object. Participants would need to move the joystick to put the object back to where they saw it in the grid during the learning phase

The procedure was kept the same between the Netherlands and China. The experiment was divided into two blocks (Fig. 1A). In each block, there was a learning phase and a recognition memory phase. Each learning phase consisted of six learning grids, during which participants were instructed to remember as many objects as possible. In the recognition memory phase, all objects in these six learning grids were tested, along with the same amount of filler objects that were not presented during this learning phase.

The current study implemented an exploration learning task (Fig. 1B; Voss, Galvan et al., 2011a, Voss, Gonsalves et al., 2011b, Voss, Warren et al., 2011c) as described in a recent study by van Lieshout et al. (2023). In each learning grid, participants were shown a 5 × 5 grid of objects covered with Gaussian noise. There was an opening (“searchlight”) that moved around to uncover the objects. Each participant was presented with six MOVE grids and six FOLLOW grids. In the MOVE condition (autonomous grids), participants were told that they could control the movement of this searchlight window by moving the joystick to explore the object grid. In a FOLLOW condition (non-autonomous grid), participants were told to follow the searchlight window (which would “move on its own”) using the joystick. This is a commonly used procedure called “yoking” (e.g., Voss, Galvan et al., 2011a, Voss, Gonsalves et al., 2011b, Voss, Warren et al., 2011c), meaning that the trajectory of the MOVE condition of the last participant was recorded and presented in the FOLLOW condition for the next participant. As such, the temporal and spatial movement of the searchlight windows were kept identical across MOVE and FOLLOW conditions. The learning task requirement was to remember as many objects as possible. The MOVE or FOLLOW condition came up one after another. The sequence of MOVE or FOLLOW grids was counterbalanced.

At the same time, REWARD or NO REWARD conditions were allocated to MOVE or FOLLOW learning grids randomly and equally between the two blocks (Fig. 1C). In each block, there would be three REWARD learning grids and three NO REWARD learning grids. In the REWARD grids, participants were told that if they remembered and successfully recognized the objects in these grids, they would get additional money (up to 5 euros in the Netherlands and 30 RMB in China) on top of the standard participation compensation. In the NO REWARD grids, participants were told that they still should try to remember these objects, but they would not get extra money for recognizing these objects.

Before each learning grid, participants would see an instruction screen indicating whether this was a MOVE (autonomous learning) or FOLLOW (non-autonomous learning) condition. In addition, for the REWARD condition, a picture of a 5-euro banknote would be presented in the middle of this instruction screen with the text (“Be aware: images from this grid are REWARDED!”) below the banknote. In China, participants would see a picture of a combination of a 20-RMB and a 10-RMB banknote with the same text. During the experiment, participants would not hear words like “volitional,” “voluntary,” or “autonomous,” but instead, they would be told that “You can move/control the window by yourself.” Each of these instruction screens before each learning grid lasted for 20 s. Participants had 60 s in each learning grid, and each learning grid was divided into two parts of 30 s. In between the two parts, participants had 20 s to rest. Each block of the learning phase lasted exactly 10 min.

In each learning block, there were three REWARD and three NO REWARD conditions. Consequently, there would be two MOVE/REWARD grids and one FOLLOW/REWARD grid in one block, whereas there would be one MOVE/REWARD grid and two FOLLOW/REWARD grids in the other block. The trajectory from a MOVE/REWARD grid would be yoked to a FOLLOW/REWARD grid to the next participant. The same was the case for the NO REWARD grids. Hence, due to the nature of the yoking procedure, the condition allocation of MOVE/FOLLOW alternated between participants. Within one block, the order of rewarded grids was randomized over the MOVE grids. The order of the rewarded follow grids was determined by the randomization over the previous (yoked-to) participant’s remaining move grids.

After every six learning grids, participants were presented with a recognition memory test, consisting of a recognition memory test and a spatial memory test. During the recognition memory test, participants were tested on the 150 objects (“old” objects) presented in the last six learning grids (Fig. 1D), as well as an equal amount of foil objects (“new” objects). In each grid of the memory test, participants had to give a response on a 4-point Likert scale using the joystick (Fig. 1D). The four possible responses were “Definitely OLD,” “Probably OLD,” “Probably NEW,” and “Definitely NEW.” If participants responded to an object as “Definitely OLD” or “Probably OLD,” participants were presented with a trial of the spatial memory test. During this test, participants were asked to put the object at the location on the grid where they saw it during the learning phase (Fig. 1D; Markant et al., 2014). In each trial of the spatial memory test, the object was initially presented in the middle of the screen with the 5 × 5 grid in the background (Fig. 1D). They could move the joystick to move the object to the correct location, and had to confirm the positioning of the object by clicking the trigger button on the joystick with their index finger. The accuracy of the spatial memory test was not considered in the additional monetary reward calculation. Participants were only instructed to try their best and to go with their best guess of the position of each object.

Participants completed 12 learning grids, during which they were presented with a total of 300 objects. They also completed two memory tests (each test took place after six grids), during which they were presented with 300 old and 300 new objects in total. At the end of the experiment, participants were informed how many objects they successfully recognized in the memory phases of the experiment (i.e., hits). They were also informed about the number of correctly recognized objects of the rewarded grids (i.e., rewarded hits) and the corresponding amount of extra monetary reward that they have won during the experiment. The calculation of the monetary rewards did not take the results of the spatial memory test into consideration. These numbers were presented on the screen.

The extra monetary reward was calculated as follows:

$$Extra\ monetary\ reward= Maximum\ monetary\ reward\times \frac{Rewarded\ hit\ objects}{Number\ of\ rewarded\ objects}$$

Before the formal experiment started, participants signed an informed consent form upon their arrival. Then, they were asked to read the instructions of the whole experiment printed on paper and explain the procedure verbally to the experimenters. This was done so that the experimenters could confirm that participants understood the task. Afterwards, participants performed a practice session, during which they were presented with four learning grids in a fixed order (one grid from each learning condition, a MOVE/NO REWARD grid, a FOLLOW/NO REWARD grid, a MOVE/REWARD grid, and a FOLLOW/REWARD grid). The pictures presented during the practice session were cartoon images (Rossion & Pourtois, 2004), so that interference of memory would not occur between the practice session and the actual experiment. Afterwards, participants completed 20 practice trials of the memory test to ensure they understood the task, including both recognition memory and the spatial memory test. Participants were instructed to try their best to remember both the objects and the locations. No data were recorded during this practice session.

### Data preparation

Data were prepared using MATLAB® R2019a (The MathWorks Inc., 2019, Natick, MA, USA). As mentioned before, participants were tested with 300 old objects (objects they had seen before) and 300 new objects (foil objects) during the memory phase of the experiment. All foil objects were deleted before the final analysis. Next, we calculated the viewing time duration of each object presented during the learning phases. Specifically, the viewing time duration was the amount of time that the searchlight window overlapped with an object picture (120 × 120 pixels) on the exploration grid. If the viewing time duration was smaller than 200 ms, this object would also be recognized as “not seen” during the learning phase. These objects would be excluded from the final analysis. Consequently, all objects that were seen by the participants during the learning phase were included in the final analysis.

After removing the filler objects in the memory test, both the Chinese dataset and the Dutch dataset consisted of a total of 13,500 recognition memory test trials (over all participants). In the Chinese dataset, we identified 450 trials in which the objects were not seen by the participants during the learning phase. Consequently, 13,050 trials from the recognition memory test were valid and included in the final analyses. For Dutch participants, 441 objects were not seen by the participants. Therefore, we included 13,059 trials from the recognition memory test in the final analysis.

We calculated three dependent variables to quantify memory performance. For the primary analyses (as reported in the main text), we focused on recognition memory (i.e., whether objects were correctly identified as old objects). To this end, the Likert responses of the seen objects were collapsed into a binary variable. For all the seen objects, if participants responded “Definitely OLD” or “Probably OLD,” they would be marked as 1 (hit). If they responded to these objects as “Probably NEW” or “Definitely NEW,” these objects would be marked as 0 (miss). Additionally, the spatial memory test performance was measured with two variables, spatial hit and spatial error. Data analysis protocols and results of spatial memory tests are reported in the Online Supplementary Material (OSM) 1.

“Hit rate” was used as the performance measure to be consistent with previous studies with a similar paradigm (Markant et al., 2014; Voss, Galvan, et al., 2011a, Voss, Gonsalves, et al., 2011b, Voss, Warren et al., 2011c). The current experimental design precluded calculating false alarms for each experimental condition. In the memory test of each block (Fig. 1A), participants were shown all learned objects in random order, intermixed with an equal number of filler objects. These filler objects could not be assigned to any of the four experimental conditions. Therefore, it is not feasible to distinguish between condition-specific false alarms, prohibiting us from calculating d’ (hit rate – false alarm) for each condition with signal detection theory (Hautus et al., 2021). However, to address the concern of group differences in response biases, d’ (hit rate – false alarm) and C (-1/2[hit rate + false alarm]) were calculated and compared between cultural groups. Details were reported in OSM 2.

### Data analysis

#### Primary analysis

We conducted linear mixed effect (LME) modelling with lme4 toolbox (Bates et al., 2015) in R (R Core Team, 2022). The dependent variable of the model was “recognition memory accuracy,” a binomial variable. The independent variables were autonomy (MOVE, autonomous learning; FOLLOW, non-autonomous learning), reward (REWARD; NO REWARD), and cultural group (CHINESE; DUTCH). Among the three factors, autonomy and reward factors were within-participant manipulations, while the cultural group was a between-participant condition. We created sum-to-zero contrasts for all the factors. In the model, we included all three main effects as fixed effects, autonomy, reward, and cultural group, respectively. The model also included two-way interaction effects between either two of these factors and the three-way interaction effect among all three factors as fixed effects. Additionally, the model had a full random effects structure, meaning that a random intercept and random slopes for all within-subject effects were included per participant (Barr, 2013; Barr et al., 2013). The LME model was fitted with 10,000 iterations and diagnosed with DHARMa (Hartig, 2020).

$$Memory\ accuracy \sim autonomy\times reward\times cultural\_group+\left(1+autonomy\times reward\;\right|sub)$$

#### Exploratory analysis

Additionally, previous findings indicated that both Chinese and Western students with higher levels of intrinsic motivation outperformed their less intrinsically motivated peers in learning tasks. However, it was found that extrinsic motivation appears to bolster learning performance only when the task performance level is low for Chinese students, who were less willing to learn (Liu et al., 2020). Moreover, a comparable result was also yielded on European students in a previous study (Murayama & Kuhbandner, 2011). It was found that for German students, their memory would only be boosted by money for boring materials, in other words, when they had no willingness to learn. These suggested that the effect of extrinsic motivation on learning may vary according to the learning performance of students or the willingness to learn. Hence, to explore the dataset, we separated each cultural group into two groups based on their performance on the recognition memory test (i.e., high achievers and low achievers). To split the participants by achievement level, we calculated the recognition memory hit rate for each participant as follows:

$$Hit\ rate=\frac{Number\ of\ hit\ objects}{Number\ of\ seen\ objects}$$

People who showed a higher or equal recognition memory hit rate than the median of their cultural group would be identified as high achievers, while people who showed a lower recognition memory hit rate than the median of their cultural group would be identified as low achievers. Consequently, we would have 23 participants in each cultural group as high achievers and 22 participants in each cultural group as low achievers. We will implement the same data analysis procedure as described for the full dataset on high achievers and low achievers separately.

## Results

The current study aimed to investigate how intrinsic and extrinsic motivation improve learning under different cultural backgrounds. In an exploratory learning task, Chinese and Dutch participants viewed partially obscured images that they needed to subsequently remember. We compared the effects of autonomy (as volitional control over the exploration trajectory) and monetary reward on the subsequent recognition memory of the objects viewed between the two cultural groups of interest.

### Primary results

**Fig. 2:**
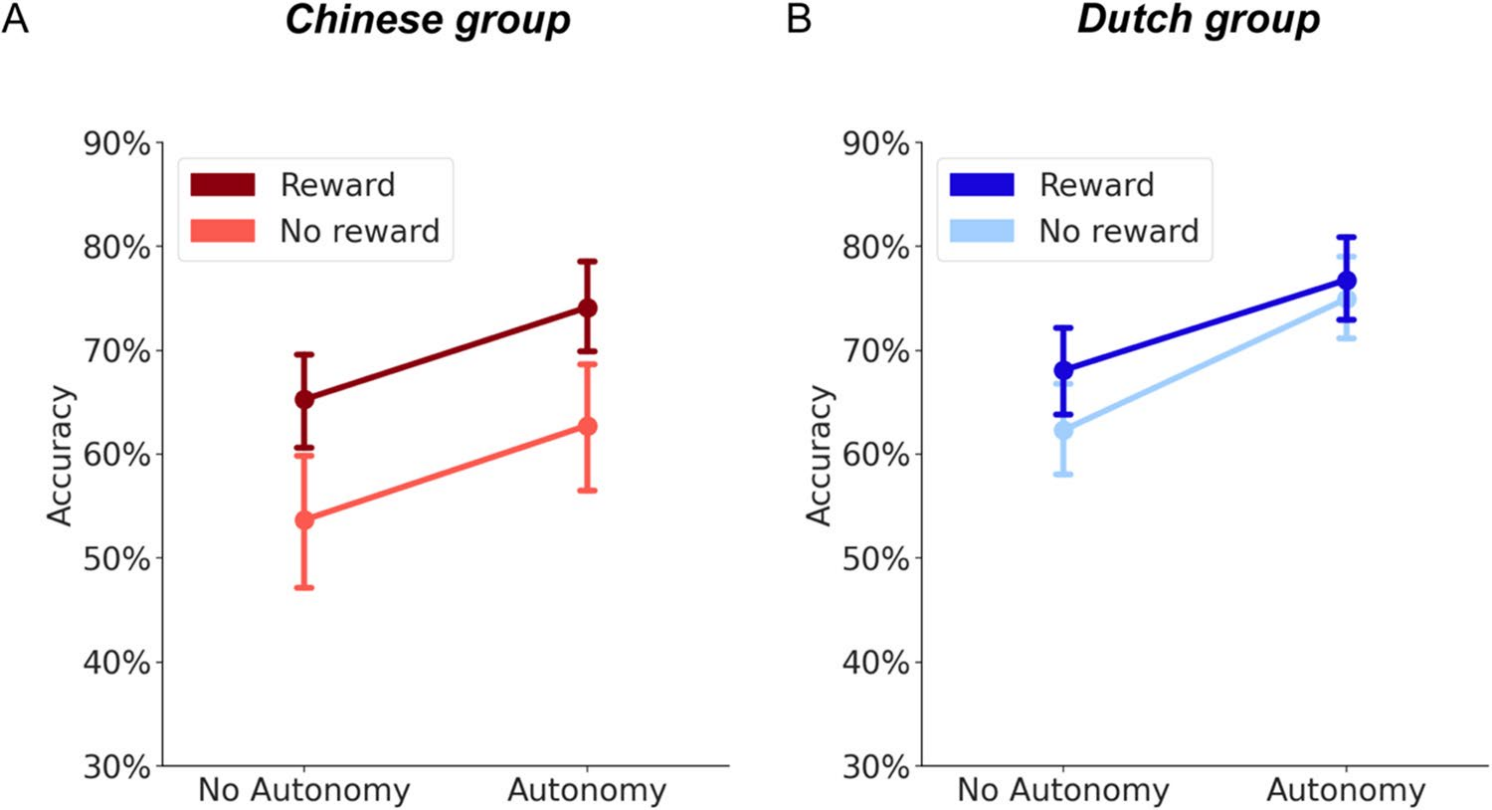
Primary results on recognition memory accuracy. Recognition memory (i.e., percentage of the correctly remembered objects) results are shown as a function of the three factors of interest: autonomy, reward, and cultural group. **A.** For the Chinese group, **r**ecognition memory is plotted as a function of autonomy and reward. The red color represents the Chinese cultural group. The dark red color represents the reward condition, while the light red color represents the no reward condition. **B.** As in **A**, recognition memory is plotted the same for the Dutch group. The blue color represents the Dutch cultural group. The dark blue color represents the reward condition, while the light blue color represents the no-reward condition. In all panels, the error bars represent the standard error of the mean. Since the three-way interaction between the factors of autonomy, reward and cultural group was not significant, we did not conduct post hoc comparisons on the two-way interaction between autonomy and reward within each cultural group

Main effects and interactions between the factors of interest, autonomy (MOVE vs. FOLLOW), monetary reward (REWARD vs. NO REWARD), and cultural group (CHINESE vs. DUTCH), were assessed in a three-way LME model on the dependent variable of recognition memory accuracy. The model results with statistics are reported in Table 1 and the data are plotted in Fig. 2. The mean and standard deviation of recognition memory accuracy for the conditions of interest are reported in Table 2.

**Table 1:** Primary results on recognition memory accuracy

Effect of interests	*Β*	*z*	*P*
Autonomy	-0.27	-8.74	<0.001***
Reward	-0.18	-5.81	<0.001***
Cultural group	-0.15	-1.81	0.41
Autonomy × Reward	-0.02	-1.09	0.29
Reward × Cultural group	-0.09	-2.85	0.004**
Autonomy × Cultural group	0.03	0.96	0.22
Autonomy × Reward × Cultural group	0.02	1.59	0.11

There are three factors included in this LME model, autonomy (MOVE/FOLLOW), reward (REWARD/NO REWARD), and cultural groups (Chinese/Dutch)

**Table 2:** Descriptive results on recognition memory accuracy

					High achievers				Low achievers			
	Chinese		Dutch		Chinese		Dutch		Chinese		Dutch	
*Main factors*	*M (%)*	*SD*	*M (%)*	*SD*	*M (%)*	*SD*	*M (%)*	*SD*	*M (%)*	*SD*	*M(%)*	*SD*
MOVE	68.74	16.04	75.90	12.84	80.74	9.71	85.11	7.78	56.19	10.84	66.27	9.58
FOLLOW	59.76	17.25	65.24	14.37	72.81	11.24	80.74	9.71	46.11	10.47	54.42	10.95
REWARD	70.04	13.76	72.63	12.19	79.36	9.22	81.45	6.33	60.30	10.63	63.40	9.73
NO REWARD	58.64	21.05	68.75	13.19	74.50	10.85	79.31	6.52	42.06	15.55	57.70	8.36
Autonomy * Reward												
MOVE/REWARD	74.23	14.36	76.78	13.54	83.22	9.44	84.83	9.55	64.84	12.58	68.36	11.97
MOVE/NO REWARD	62.97	20.93	74.96	13.57	78.12	11.26	85.34	7.15	47.14	16.47	64.11	9.53
FOLLOW/REWARD	65.39	15.45	68.08	14.91	74.86	12.30	77.90	8.37	55.49	11.87	57.81	13.28
FOLLOW/NO REWARD	54.11	22.44	62.31	15.68	70.67	12.34	73.23	9.96	36.80	16.77	50.90	12.00

**Fig. 3:**
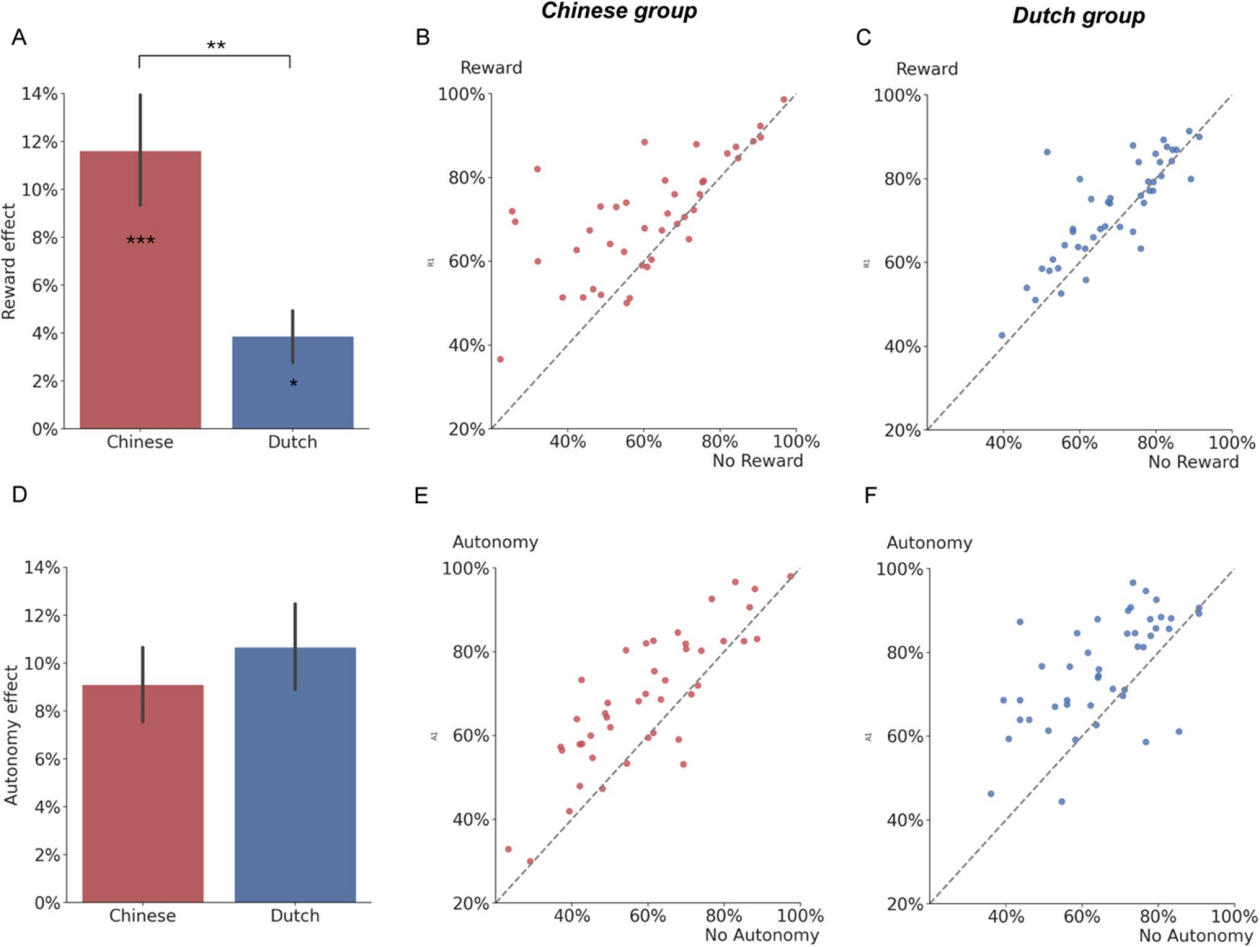
Individual variability in the beneficial effect of reward and autonomy based on recognition accuracy. In all graphs, the red color represents the Chinese cultural group, while the blue color represents the Dutch cultural group. **A.** The beneficial effect of reward on memory accuracy (REWARD – NO REWARD) is stronger for the Chinese group than for the Dutch group. The bars (y-axis) represented the beneficial effect of reward on recognition memory. The error bars represent the standard error of the mean of reward effect. Asterisks on the bars represent the significance of the beneficial effect of reward on the recognition memory accuracy of each group. ***p < 0.001; *p < 0.05. **B.** Chinese group individual variability in mean recognition memory accuracy for the REWARD condition (y-axis) compared with the NO REWARD condition (x-axis). Each dot represents a participant. Most dots tend to lie above the diagonal, illustrating that most of the Chinese participants had a higher recognition memory accuracy in the REWARD condition than in the NO REWARD condition. **C.** Dutch group individual variability in mean recognition memory accuracy for the REWARD condition (y-axis) compared with the NO REWARD condition (x-axis). Each dot represents a participant. While the dots lie close to the diagonal, more dots still lie above the diagonal. This illustrates the significant but smaller beneficial effect of reward on recognition memory than in the Chinese group. **D.** The beneficial effect of autonomy on learning did not differ between the Chinese and Dutch groups. The bars (y-axis) represent the beneficial effect of autonomy (MOVE – FOLLOW) on recognition memory. The error bars represent the standard error of the mean**. E.** Chinese group individual variability in mean recognition memory accuracy for the MOVE condition (y-axis) compared with the FOLLOW condition (x-axis). Each dot represents a participant. Most dots tend to lie above the diagonal, illustrating that most of the Chinese participants had a higher recognition memory accuracy in the MOVE condition than in the FOLLOW condition. **F.** Dutch group individual variability in mean recognition memory accuracy for the MOVE condition (y-axis) compared with the FOLLOW condition (x-axis). The distribution of the dots is similar to Fig. 3E, suggesting a similar beneficial effect of autonomy on learning between two cultural groups

We found a main effect of autonomy and reward on recognition memory accuracy (Table 1). This indicated that participants learned better in the MOVE condition than in the FOLLOW condition. Also, participants learned better in the REWARD condition than in the NO REWARD condition. We did not find a main effect of cultural group on recognition memory accuracy. This suggested that Dutch students had a similar performance to Chinese students in the recognition memory test. For the two-way interaction effects of interest, we did not find an interaction between autonomy and reward on recognition memory accuracy. This indicated that, if we view the two cultural groups as one sample, the beneficial effect of autonomy on memory would not be affected by external rewards (Fig. 2). Interestingly, we found a significant two-way interaction effect between factors of reward and cultural group on recognition memory accuracy (Figs. 3A–C). We did not find a two-way interaction effect on recognition memory accuracy between factors of autonomy and cultural group (Figs. 3D–F). This suggested that the beneficial effect of autonomy on recognition memory accuracy was similar between the Chinese and Dutch cultural groups. We also did not find a significant three-way interaction among autonomy, reward, and cultural groups Table 3.

**Table 3:** Statistical results of recognition memory accuracy from high achievers and low achievers

	High achievers			Low achievers		
Effect of interests	*β*	*t*	*p*	*β*	*t*	*p*
Autonomy	-0.29	-6.32	<0.001***	-0.24	-6.06	<0.001***
Reward	-0.11	-3.64	<0.001***	-0.27	-5.26	<0.001***
Cultural group	-0.09	-1.10	0.29	-0.21	-3.64	0.002**
Autonomy × Reward	-0.02	-0.74	0.50	-0.02	-0.92	0.36
Reward × Cultural group	-0.04	-1.53	0.16	-0.14	-2.76	0.005**
Autonomy × Cultural group	0.04	0.88	0.16	0.02	0.41	0.66
Autonomy × Reward × Cultural group	0.05	2.06	0.04*	0.01	0.26	0.80

There are three factors included in the LME models above, autonomy (MOVE/FOLLOW), reward (REWARD/NO REWARD), and cultural groups (Chinese/Dutch)

To disentangle the interaction between reward and cultural group further, we compared memory accuracy for the REWARD and NO REWARD conditions, respectively, for the Dutch group and the Chinese group (Fig. 3A) with the *emmeans* package in R (Lenth, 2022). It was found that the facilitatory effect of reward (REWARD – NO REWARD) on recognition memory was significant for both the Chinese group (*β* = 0.55, *z* = 6.14, *p* < 0.001) and the Dutch group (*β* = 0.19, *z* = 2.11, *p* = 0.03). Moreover, this reward effect on memory was found to be stronger for the Chinese group compared with the Dutch group (Fig. 3A). This difference between cultural groups is also apparent when plotting the reward effects of each participant in the Chinese (Fig. 3B) and Dutch group (Fig. 3C). Alternatively, we also compared recognition memory accuracy between the Chinese and Dutch groups under both REWARD and NO REWARD conditions, respectively (Fig. 2). It was found that under REWARD conditions, the Dutch group and the Chinese group performed similarly (*β* = -0.11, *z* = -0.73, *p* = 0.47) in the recognition memory test. However, under the NO REWARD condition, the Dutch group performed better than the Chinese group (*β* = -0.47, *z* = -2.48, *p* = 0.01) in the recognition memory test.

For completeness, we also plotted the autonomy effect between cultural groups (Fig. 3D). The individual variability of the autonomy effect on memory accuracy for the Chinese group is plotted in Fig. 3E. The same is shown for the Dutch group in Fig. 3F.

### Exploratory results: High achievers and low achievers

Additionally, we performed exploratory analyses to investigate whether the reported primary results differ based on participants’ task performance. This was done because previous research has indicated that extrinsic motivation appeared to improve learning performance among Chinese students when their task performance was initially suboptimal (Liu et al., 2020). However, for Western students, extrinsic motivation equally boosted learning for students regardless of task performance. This suggested that the reported effect of extrinsic motivation on learning might be modulated by both cultural group and task performance. To this end, we divided the Chinese and Dutch cultural groups into “high achievers” and “low achievers,” and applied the same model used for the primary analysis to the high- and low-achiever groups separately.

**Fig. 4:**
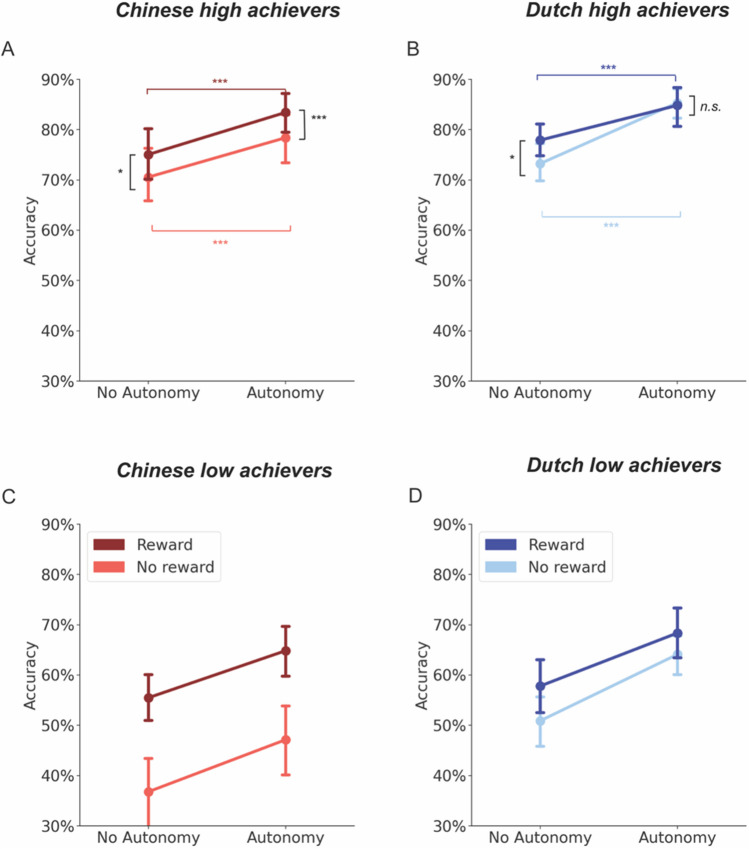
Results on recognition memory accuracy after splitting each cultural group into high and low achievers. **A.** For the Chinese high achievers, recognition memory is plotted as a function of autonomy and reward. The red color represents the Chinese high achievers. The dark red color represents the reward condition, while the light red color represents the no reward condition. The colored lines represent the effect comparison (MOVE FOLLOW) under REWARD or NO REWARD conditions. Asterisks near the red comparison lines indicated the significance of (MOVE – FOLLOW) under different reward conditions. Asterisks next to the black comparison lines indicated the significance of (REWARD – NO REWARD) under different autonomy conditions. The error bars represent the standard error of the mean (*SEM*). (***:* p* < 0.001; *: *p* < 0.05). **B.** For the Dutch high achievers, recognition memory is plotted the same. The blue color represents the Dutch cultural group. The dark blue color represents the REWARD condition, while the light blue color represents the NO REWARD condition. The rest of the conventions were the same as in Fig. 4A (***:* p* < 0.001; *: *p* < 0.05; *n.s*.: *p* > 0.05). **C.** Recognition memory accuracy for Chinese low achievers. All conventions are the same as in Fig. 4A. **D.** Recognition memory accuracy for Dutch low achievers. All conventions are the same as in Fig. 4B

When focusing on the high achievers only, we found a significant three-way interaction between the factors of autonomy, reward, and cultural group on recognition memory accuracy. To dig deeper into this three-way interaction, we compared the recognition memory accuracy between the two cultural groups under each condition of reward and autonomy with *emmeans* package in R (Lenth, 2022). We did not find significant differences between Chinese and Dutch high achievers (Chinese – Dutch) under the MOVE/REWARD condition (*β* = -0.08, *z* = -0.34, *p* = 0.74), FOLLOW/REWARD condition (*β* = -0.11, *z* = -0.58, *p* = 0.56), and FOLLOW/NO REWARD condition (*β* = -0.09, *z* = -0.49, *p* = 0.63). However, only for the MOVE/NO REWARD condition, we found that the Chinese high achievers exhibited a lower recognition memory accuracy than the Dutch high achievers (*β* = -0.44, *z* = -2.19, *p* = 0.03). Additionally, we also compared the reward effect on recognition memory accuracy (REWARD – NO REWARD) under either MOVE or FOLLOW conditions for each cultural group separately. For Chinese students, we found that reward improved learning under both MOVE (*β* = 0.36, *z* = 3.33, *p* = 0.001) and FOLLOW (*β* = 0.24, *z* = 2.36, *p* = 0.02) conditions (Fig. 4A). For Dutch participants, however, extra rewards only improved learning under the FOLLOW condition (*β* = 0.26, *z* = 2.57, *p* = 0.01), not under the MOVE condition (*β* = 0.01, *z* = 0.06, *p* = 0.96; Fig. 4B).

Secondly, when focusing on the low achievers, we found a significant two-way significant interaction effect between reward and cultural group on memory accuracy (Figs. 4C and D). This was consistent with the results of the primary analysis. When breaking down this interaction effect, it was found that the facilitatory effect of reward on memory accuracy was larger for the Chinese low achievers (*β* = -0.83, *z* = -5.66, *p* < 0.001) than for the Dutch low achievers (*β* = -0.26, *z* = -1.77, *p* = 0.08). Alternatively, we also found that, under the REWARD condition, Dutch and Chinese low achievers performed similarly (*β* = -0.14, *z* = -1.01, *p* = 0.31). However, under the NO REWARD condition, Dutch low achievers performed better in the recognition memory test than Chinese low achievers (*β* = -0.71, *z* = -4.11, *p* < 0.001).

To summarize, we found that reward improved memory accuracy for Dutch high achievers under the FOLLOW condition (no autonomy), but not under the MOVE condition. However, for Chinese high achievers, the reward effect was present for both MOVE and FOLLOW conditions. Meanwhile, Chinese low achievers were motivated to learn by monetary rewards more compared with Dutch low achievers. It was also evident that Chinese low achievers only performed less effectively compared to Dutch low achievers when without rewards.

## Discussion

In our study, we delved into the impact of intrinsic and extrinsic motivation on learning across diverse cultural contexts, by focusing on the comparisons between Chinese and Dutch student populations. Participants engaged in an exploratory learning activity where they were presented with partially obscured images, which they were required to recall later. We manipulated autonomy (representing intrinsic motivation) by granting participants control over their exploration trajectory, and we also varied the opportunity for monetary rewards (representing extrinsic motivation) independently. Throughout the experiment, participants were tasked with memorizing as many objects as possible, followed by a subsequent memory assessment. By administering the same learning experiment to Chinese and Dutch students, the current study aimed to gain a better understanding of the cultural differences in intrinsic and extrinsic motivation for learning.

There are three key novel findings in this experiment. First, we found that the beneficial effect of extrinsic motivation (i.e., monetary reward) on learning was stronger for Chinese students than for Dutch students (e.g., Zhu & Leung, 2011). Second, we found that there was no difference in the beneficial effect of intrinsic motivation (i.e., autonomy) on learning between Chinese and Dutch students (e.g., Ryan & Deci, 2006). Third, when including all participants, we did not find an interaction effect between autonomy and reward on learning in either cultural group, which differs from previous studies (e.g., van Lieshout et al., 2023). However, in an exploratory analysis taking learning achievement into account, we found that for Dutch high achievers, the beneficial effect of reward was diminished in autonomous learning compared to non-autonomous learning conditions (Fig. 4B; van Lieshout et al., 2023). In contrast, reward improves learning regardless of autonomy for Chinese high achievers (see Fig. 4A). These results together support the idea that intrinsic motivation for learning may be culturally universal, while extrinsic motivation for learning is stronger for Chinese students than for Dutch students. Furthermore, the interaction effect between intrinsic and extrinsic motivation on learning needs to be discussed with regard to different cultural groups and concerning different levels of learning outcomes.

### The effect of reward on learning was stronger for Chinese than for Dutch students

In both cultural groups, participants remembered more objects in the REWARD condition than in the NO REWARD condition. However, Chinese students exhibited a stronger effect of reward on memory than Dutch students, indicated by a significant interaction effect between factors of reward and cultural group (Fig. 3A). When delving deeper into this interaction effect, it was found that Chinese students remembered fewer objects compared with Dutch students when there was no monetary reward. Students from the two cultural groups performed equally well for the rewarded objects.

This is consistent with findings from previous studies suggesting that people from a collectivistic cultural background would be more motivated by external sources (Huang, 2013). In our current setting, one of the goals was to obtain extra monetary rewards. However, the goals participants pursue do not necessarily have to consist of monetary rewards (e.g., Huang, 2013; Zhu & Leung, 2011); they can also encompass group benefits (Salili et al., 2012), or achievements (Telzer et al., 2017). As distinct from Western philosophy, Chinese cultural contexts emphasize academic success and attainment (Dekker & Fischer, 2008). The pursuit of education is traditionally intertwined with collective aspirations, such as upholding family honor and contributing to the broader society (Salili et al., 2012). This ethos stems from the Confucian principle of "Rushi" (入世), which promotes self-improvement and contribution to societal prosperity (Hao, 2018). In Confucian culture, factors that come from external environments are more strongly emphasized than in non-Confucian Western educational contexts, like materialistic rewards, academic achievement, expectancy of success, and group benefits (Blevins et al., 2023; Chen et al., 2005; Iyengar & DeVoe, 2003; Telzer et al., 2017). Students with Confucian cultural backgrounds develop an intrinsic passion and commitment to learning after understanding the importance of learning in life-building and self-development (e.g., Liu et al., 2020), while in Western culture, learning is usually driven by interest. Furthermore, after separating participants into high and low achievers, it was observed that the cultural difference in the beneficial effect of reward on memory only existed for low achievers, not for high achievers. This is also in agreement with previous findings suggesting that students with a Confucian cultural background and low performance in learning showed a higher sense of extrinsic motivation for learning (Liu et al., 2020).

Specifically, Eastern culture deems norms of extrinsic motivation as more meaningful and essential compared with Western culture (Tao & Hong, 2013), shaping reward circuitry activity underlying specific behaviors. From the sociocultural brain perspective, neural responses toward external stimuli are shaped by both short- and long-term dynamic cultural experiences (Han, 2017; Han et al., 2013). For instance, previous studies have found that cultural backgrounds shape the activation of the ventral striatum toward monetary rewards (Kim et al., 2012). People with Eastern cultural backgrounds would have persistent reward circuitry activation even when the reward is delayed. Moreover, compared with American participants, Chinese participants showed more sustained reward circuitry activation (in the ventral striatum) during a go/no-go task when their goal was to improve their accuracy in this task (Telzer et al., 2017). In this situation, Chinese students were more motivated by gaining higher task achievement than American students were. This observation is consistent with the cultural valuation of achievement, which is notably higher for Chinese students compared to Western students (Tao & Hong, 2013). Integrating our findings and the sociocultural brain perspective (Han, 2017; Han et al., 2013), culture plays a critical role in shaping one's sensitivity towards various motivational factors, which is closely tied to the functioning of the reward system. In contrast, the cultural influences might not extend to the biological underpinnings of the reward system, such as dopamine receptors (Glazer et al., 2020).

Interestingly, there was a study specifically indicating that monetary reward does not cause different levels of activation on reward circuitry between different cultural groups (Blevins et al., 2023). However, it is crucial to emphasize that upon closer examination of their results, our current findings are in alignment with theirs. Although in their study there were no differences in reward circuitry activation between Chinese and American groups when they received monetary rewards, American participants showed higher nucleus accumbens (NAcc) activity compared to Chinese participants when they received NO monetary rewards during the target-hitting task (Blevins et al., 2023; Supplementary Material, Section 11, page 26). These findings resonate with the results presented in the current study, as we observed that Chinese participants demonstrated weaker recall for objects that were not rewarded in comparison to Dutch participants. However, this discrepancy was absent when rewards were involved. Hence, we hypothesize that cultural norms can shape functional engagement of certain brain systems during the learning phase in the absence of rewards. From the perspective of neuroplasticity that is formed due to learning of culture norms (Han, 2017; Han & Ma, 2014), Chinese students might tend to use relatively more external-driven strategies during learning, leading them to exhibit a lower baseline activation in reward circuitry when they are learning for NO external drives or purposes. However, this hypothesis requires future research to be substantiated.

In summary, extrinsic motivation is universally recognized for enhancing behavioral performance. This is likely due to the regulatory effect of extrinsic motivation on activity in the reward circuitry (e.g., striatum). Our study further clarifies that this effect is more pronounced in Chinese individuals compared to Dutch individuals during learning tasks, suggesting cultural variability in cognitive and neural responses to extrinsic motivators.

### The beneficial effect of reward on learning can be affected by context

In the current study, we found that only for Dutch high achievers was the effect of reward on learning not present when their intrinsic motivation (autonomy) was invoked. However, the reward effect on learning always existed for Chinese high achievers. This finding aligned with the previous notion that the interaction between intrinsic and extrinsic motivation in learning is not always present and has been over-generalized (Eisenberg, 2002). One possible interpretation of the diminishing reward effect in Dutch high achievers with autonomy is that they do not need rewards to heighten their motivation, because autonomy as an intrinsic motivator is already sufficient (Cameron, 2001). Similar to results from Murayama and Kuhbandner (2011), when German students were learning interesting content (with intrinsic motivation to learn), money does not boost learning performance. Instead, money only improved learning when the content was boring. This notion is also supported by our findings, such that Dutch high achievers performed better than Chinese high achievers when they were learning autonomously but were not rewarded for their performance. However, their learning performance was equally high when both autonomy and rewards were provided. To rephrase, autonomy alone may suffice as a significant motivational driver for Dutch high achievers, enabling them to learn to the best of their ability. Conversely, for Chinese high achievers, the presence of autonomy does not fully maximize their motivational potential for learning, indicating that their learning motivation has not yet reached its peak.

An alternative interpretation is that the effect of autonomy is diminished in the presence of rewards compared to the absence of rewards for Dutch high achievers. This could be caused by the fact that Dutch high achievers perceived extrinsic rewards as controlling, which stands in stark contrast to experiencing autonomy during learning. Therefore, the advantageous impact of autonomy on the learning process is potentially diminished (i.e., overjustification; Hidi, 2015; Lepper et al., 1973). This is in line with educational studies indicating that extrinsic motivation is detrimental for academic achievement for Western students, while both intrinsic and extrinsic motivators are beneficial for Chinese students (Zhu & Leung, 2011).

Additionally, we also found that the beneficial effect of rewards on learning was stronger for Chinese students, but only for low achievers (Figs. 4C and D). This discovery aligns with the findings of prior research, suggesting that the influence of rewards on performance might be modulated by levels of achievement (Liu et al., 2020). On the contrary, there are recent studies suggesting that the effect of rewards on behavioral performance is stronger for Western than for Eastern culture (Liu et al., 2020; Medvedev et al., 2024). This was likely caused by the nature of their measurements, which were imbued with social or external values (i.e., helping the researcher to build up a machine-learning model or learning math). As we stated before, different fragments of motivation are stated and perceived as more meaningful in different cultural backgrounds. For instance, in the setting of Medvedev et al. (2024), a sense of relatedness (i.e., one of the components that foster intrinsic motivation, according to self-determination theory) was induced. Relatedness, defined as a feeling of connection with others, might be more meaningful for Chinese culture than for Western culture (e.g., Walker et al., 2020). Therefore, when relatedness is elicited, Chinese participants might rely less on additional extrinsic motivators than Western participants. This supports our claim that various intrinsic motivators can affect extrinsic motivation differently, depending on the cultural context.

Taken together, in line with the sociocultural brain perspective, for high achievers with Dutch cultural backgrounds, intrinsic motivation (i.e., autonomy) can reduce the effectiveness of extrinsic motivation on learning outcomes and vice versa. However, this interaction effect between intrinsic and extrinsic motivation on learning did not exist for Chinese participants nor for Dutch low achievers. This highlights the mutual influence of various motivators throughout the learning process. Our findings align with and extend the sociocultural brain perspective (Han et al., 2013), highlighting that learning motivation is shaped not only by the cultural environment but also by levels of achievement in learning contexts.

### Autonomy improved learning in both cultural groups

Furthermore, we did not find cultural differences in the beneficial effect of intrinsic motivation on learning. This was indicated by the strong effects of autonomy on memory performance, which were present for both Chinese and Dutch students. These findings are congruent with the assertions of SDT, which posits that autonomy is a fundamental psychological need and, akin to biological drives, is a universal phenomenon across different cultures (Helwig, 2006; Ryan & Deci, 2017). Moreover, intrinsic motivation, particularly autonomy, is closely linked to the pursuit of personal challenges (Di Domenico & Ryan, 2017). While intrinsic motivation and self-improvement are often highlighted in Western ideologies, these concepts are also deeply valued in Eastern tradition. For instance, Confucian philosophy emphasizes the importance of self-cultivation and personal reflection (Zusho, 2005), and Taoism emphasizes the sense of autonomy and freedom in personal behaviors (Wenzel, 2003).

Our study also corroborates neuroscientific evidence that both Chinese and Western individuals exhibit strong motivational brain responses linked to autonomy. For example, in both cultures, feedback-related negativity was stronger for self-relevant rewards compared to rewards relevant for others (Kitayama & Park, 2014; Zhu et al., 2016). Similarly, increased activation in the medial prefrontal cortex and anterior cingulate cortex is observed during self-related personality judgment tasks among participants from both Chinese and Western cultural backgrounds (Zhu et al., 2007). In our current setup, when participants were autonomously exploring the grid with objects, their personal connection to those objects was likely heightened. This might result in stronger brain activity in the dorsal lateral prefrontal cortex (DLPFC) under autonomous conditions, thereby improving learning outcomes across diverse cultural backgrounds.

From a neuroscientific perspective, intrinsic motivation, like autonomy, might trigger not only activation and connectivity among a network of distributed brain regions including the OFC and VMPFC, subcortical dopaminergic circuitry, and hippocampus, but also enhance engagement of the DLPFC, which is associated with attentional control (Voss, Gonsalves, et al., 2011b). In contrast, extrinsic motivation, like monetary rewards, tends to specifically engage modulation from VMPFC and dopaminergic circuitry (e.g., VTA) influencing the hippocampus (Adcock et al., 2006; Wolosin et al., 2012). Combined with our current behavioral findings, we could hypothesize that cultural background shaped functional activation and connectivity among distributed regions including VMPFC, dopaminergic circuitry, and hippocampus of Eastern students to be more sensitive to rewards in learning tasks. However, with intrinsic motivation exerted on top of extrinsic motivation, DLPFC becomes engaged with a higher level of attentional control over this reward-related brain network, diminishing the cultural difference in intrinsic motivation. Interestingly, this also aligned with a meta-analysis on brain activity focusing on social cognitive processes, for instance, self-reflection tasks where people rate descriptions of their personalities. They found that East Asians exhibited a stronger activity in DLPFC, while Westerners exhibited stronger activation in VMPFC (Han & Ma, 2014) in these social cognitive processes.

Taken together, our behavioral findings might shed light on both overlap (i.e., VMPFC, OFC, reward circuitry, the hippocampus) and potential dissociations (i.e., DLPFC) of the brain mechanism of intrinsic and extrinsic motivations in learning.

### Future directions and limitations

In the end, there is still a lack of studies investigating brain mechanisms underlying the overlap, distinctions, and interactions between extrinsic motivation and intrinsic motivation in learning, particularly regarding the modulation of this process by individual differences. In the future, conducting the current behavioral study in conjunction with functional magnetic resonance imaging (fMRI) could provide valuable insights into the neural underpinnings of cultural differences affecting the interplay between extrinsic and intrinsic motivation in learning. To start with, our current findings, combined with previous neuroimaging studies, indicated that there might be cultural differences in both their behavioral performance and brain activation when participants are extrinsically motivated to learn. Previous research has shown that extrinsic motivation elicits more connectivity among VMPFC, midbrain, VTA, and hippocampus (e.g., Adcock et al., 2006). We hypothesize that the beneficial effect of reward on this brain connectivity would be stronger for Chinese students compared to Dutch students. Second, we found that autonomy could enhance learning equally across cultural groups. Furthermore, Voss, Galvan et al. (2011a), Voss, Gonsalves et al. (2011b), Voss, Warren et al. (2011c) found that autonomous control (intrinsic motivation) could provoke connectivity between the hippocampus and brain areas related to attentional coordination, like the DLPFC. Hypothetically, this brain connectivity between the DLPFC and hippocampus might remain the same across cultural groups. Third, we found that the interaction effect between intrinsic and extrinsic motivation on learning does not uniformly apply across all participants. Regarding cultural differences in motivation, research indicated that Western individuals showed increased activity in brain regions related to both attentional control (i.e., DLPFC) and reward circuitry (i.e., VTA) during experiences of self-control. Conversely, Eastern individuals demonstrated similar brain activation patterns in scenarios where they felt under control by others (Freeman et al., 2009). As we discussed before, cultural backgrounds may shape individuals to perceive varying motivators as more meaningful. This revelation suggests that both the reward circuitry and the prefrontal cortex, related to different types of motivation, might be activated differently depending on cultural context. Hence, it would be intriguing to utilize the current design in an fMRI study to explore motivation-related connectivity among reward circuitry, the prefrontal cortex, and the hippocampus across cultures.

Regarding limitations, the current study did not collect questionnaires assessing the cultural norms and values of each participant. Therefore, we were unable to analyze which specific cultural perspectives might have contributed to the observed differences in learning motivation between cultural groups. Future research in this area should consider incorporating such assessments to expand our understanding of these cultural attributions.

## Conclusions

To summarize, our study yielded three significant insights. Firstly, extrinsic motivation was more beneficial for learning in Chinese compared with Dutch students. Secondly, intrinsic motivation positively impacted learning across both Western and Eastern cultures. Thirdly, while extrinsic motivation did not enhance learning for high-achieving Dutch students when their intrinsic motivation was fulfilled, it always enhanced learning for low-achieving Dutch students. In contrast, extrinsic motivation consistently improved learning for Chinese students, irrespective of their performance level. These outcomes enhance our comprehension of how cultural nuances affect our motivation to learn and underscore the importance of considering these differences in educational strategies.

## Supplementary Information

Below is the link to the electronic supplementary material.

Figure S1 (PNG 110 kb)

High Resolution Image (TIFF 2539 kb)

Figure S1 (PNG 549 kb)

High Resolution Image (TIF 103856 kb)

Figure S1 (PNG 954 kb)

High Resolution Image (TIF 197617 kb)

Supplementary Material 1 (DOCX 10681 kb)

Supplementary Material 2 (DOCX 17 kb)

Supplementary Material 3  (DOCX 20 kb)

Supplementary Tables (XLSX 22 kb)

## Data Availability

All materials, data, and code used for the experimental procedure and data analyses are freely available on the Radboud Repository (10.34973/tccj-j019).
