# Supplementary figures and images for: A cross-cultural comparison of intrinsic and extrinsic motivational drives for learning
Source: Cogn Affect Behav Neurosci. 2024 Oct 18;25(1):25–44. doi: 10.3758/s13415-024-01228-2 (PMC11805854; doi:10.3758/s13415-024-01228-2)

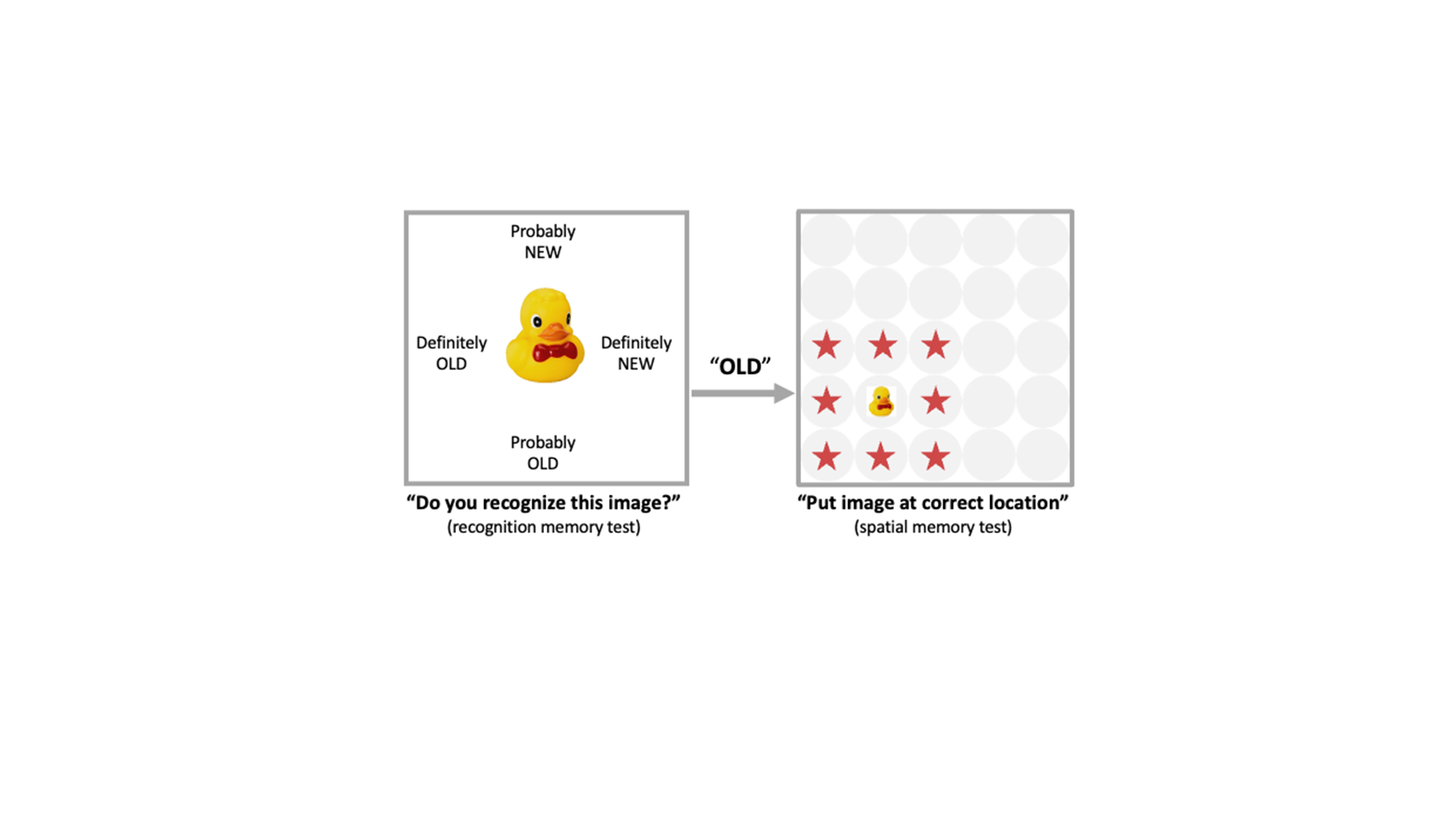

Supplement: Supplementary file 1 — (PNG 110 kb) [file 13415_2024_1228_Fig5_ESM.png]

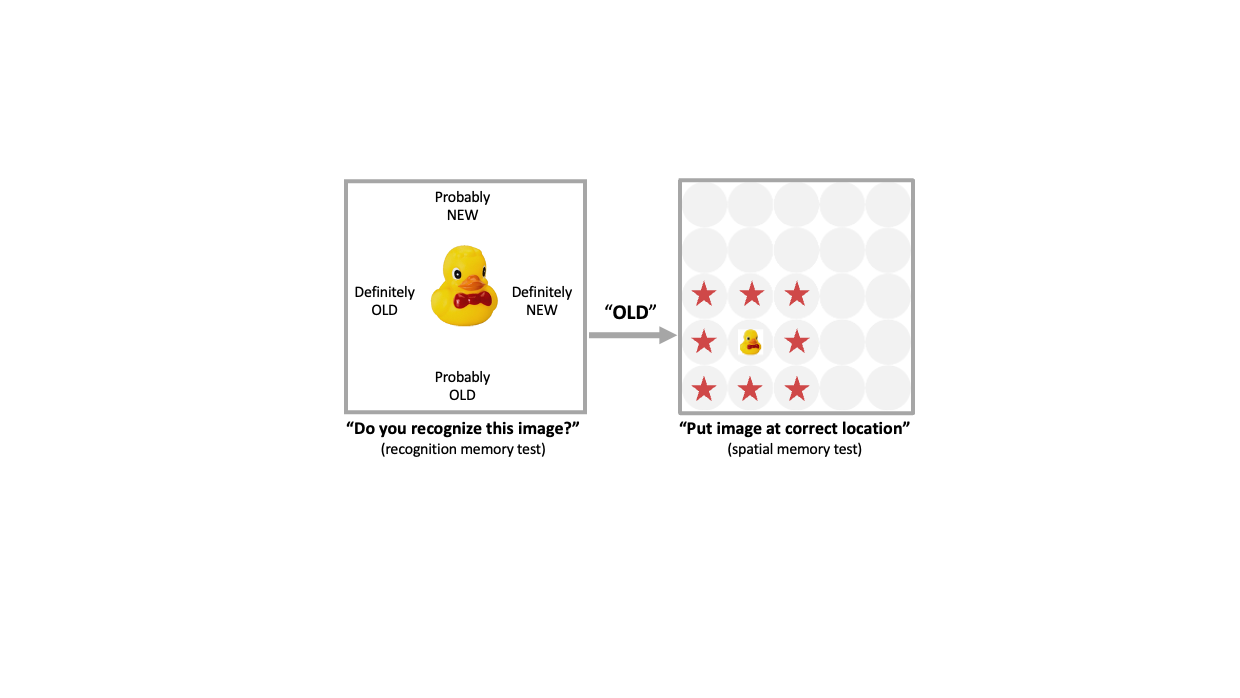

Supplement: Supplementary file 2 — High Resolution Image (TIFF 2539 kb) [file 13415_2024_1228_MOESM1_ESM.tiff]

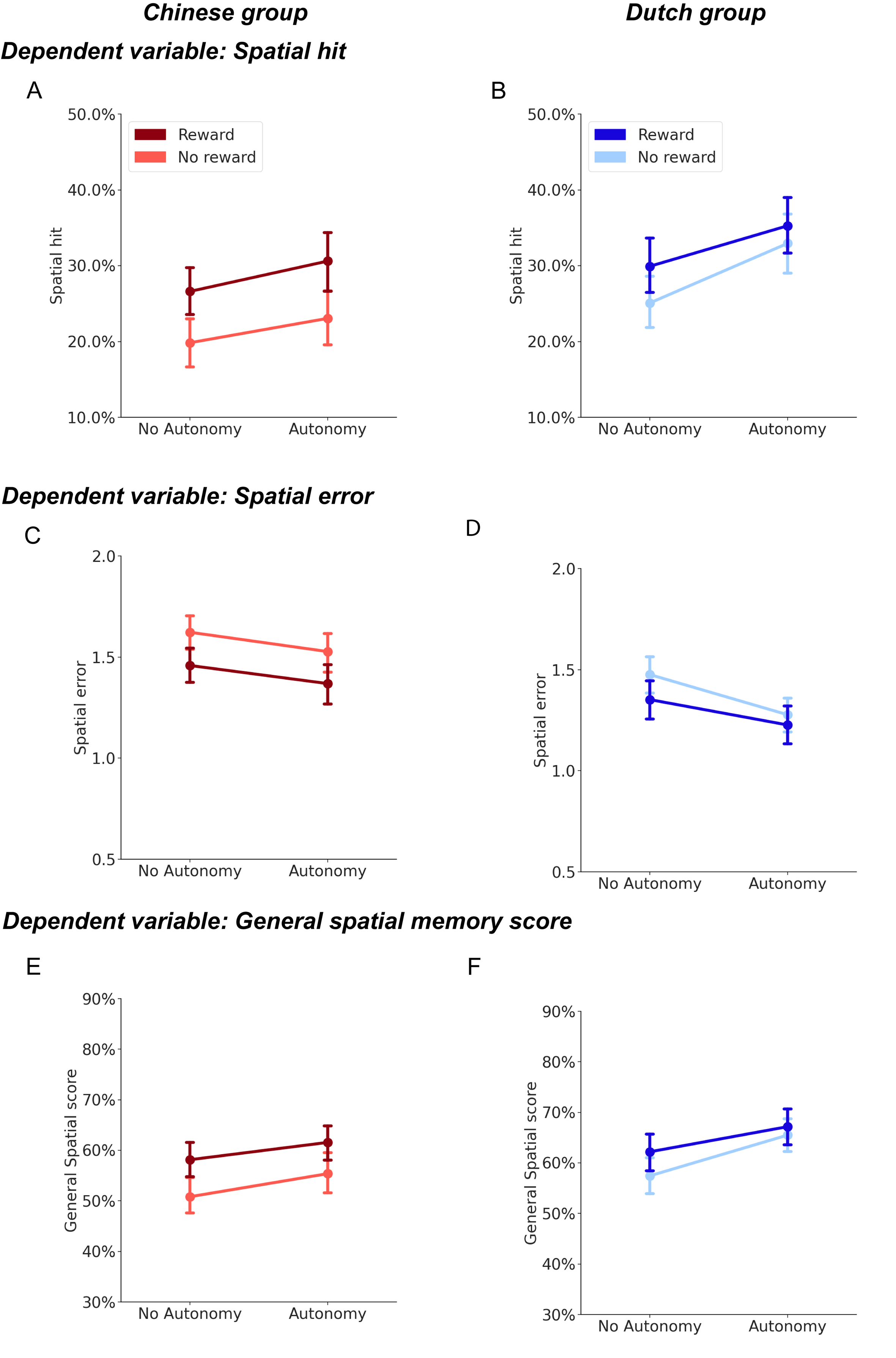

Supplement: Supplementary file 3 — (PNG 549 kb) [file 13415_2024_1228_Fig6_ESM.png]

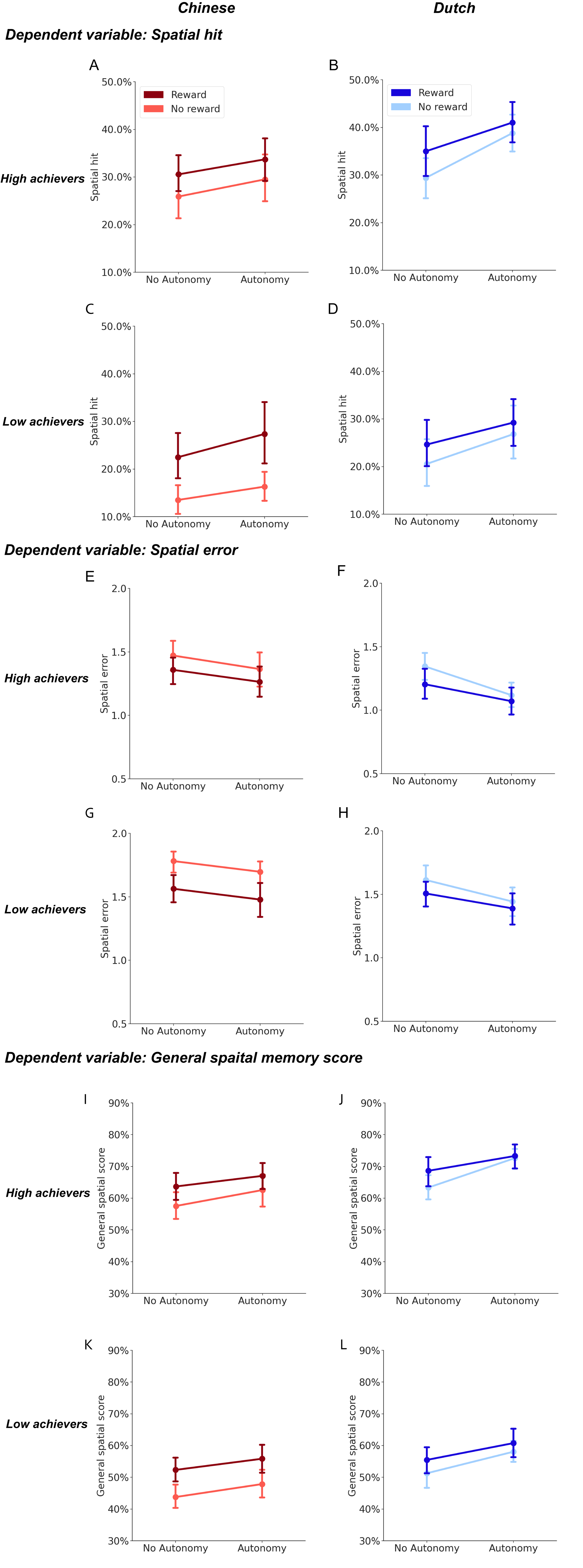

Supplement: Supplementary file 5 — (PNG 954 kb) [file 13415_2024_1228_Fig7_ESM.png]
