# Supplementary material for: A cross-cultural comparison of intrinsic and extrinsic motivational drives for learning
Source: Cogn Affect Behav Neurosci. 2024 Oct 18;25(1):25–44. doi: 10.3758/s13415-024-01228-2 (PMC11805854; doi:10.3758/s13415-024-01228-2)
Supplement: Supplementary file 7 — (DOCX 10681 kb) [file 13415_2024_1228_MOESM4_ESM.docx]

**Supplementary Material 1: Spatial memory results**

**Methods**

In the current experiment, during each trial of the memory test in which participants responded to an object as “Definitely OLD” or “Probably OLD”, they were additionally asked to put the object at the location where they saw it during the learning phase. This part of the memory test was called the spatial memory test. In this spatial memory test, there were three dependent variables calculated to represent the spatial memory performance, the spatial hit, the spatial error, and a general spatial test score.

The spatial hit was calculated and used as a binomial dependent variable, like the recognition accuracy described in the primary results. If participants managed to correctly put the object back to where they saw it during the learning phase, that trial would be counted as a “hit” (spatial hit as 1) in the spatial memory test. On the contrary, if they put it to a different location from the correct one, we counted that trial as a “miss” (spatial hit as 0).

Additionally, a spatial error was calculated, defined as the Euclidian distance between the responded location and the correct location. Hence, spatial error was a continuous variable.

$Spatial error= \sqrt{{(X_{respond}-X_{correct})}^{2}+{(Y_{respond}-Y_{correct})}^{2}}$

We applied a logarithmic transformation to the spatial errors (after adding 1 to each value to avoid 0 in this variable) to achieve a normal distribution for the dependent variable in our inferential statistical analysis.

Last, we calculated a general spatial memory score by labeling objects where participants put back to the correct location and all 8 locations surrounding that correct location (Figure S1) as “hit” (general spatial memory score as 1). On the contrary, if they put the object to the rest 16 locations from these 9 locations, we counted that trial as a “miss” (general spatial memory score as 0).


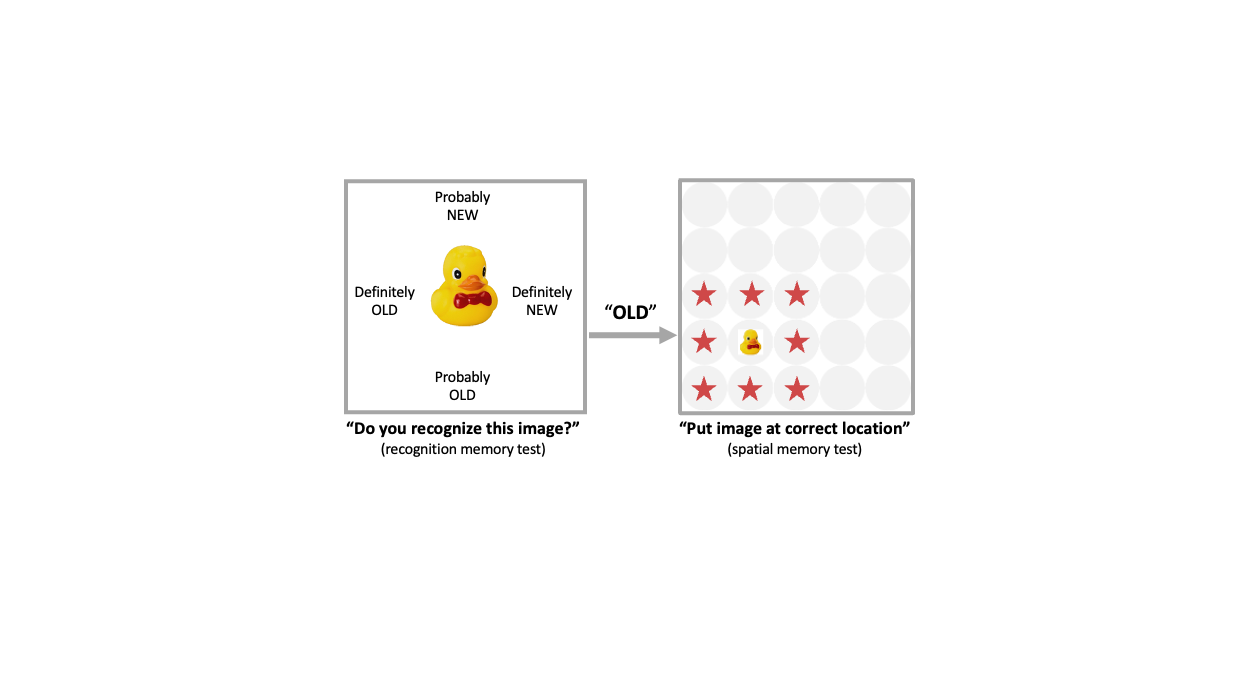


**Figure S1 General spatial memory score calculation**

In the memory test, participants were asked to put the OLD objects back to the locations they saw the object during learning. When computing the general spatial memory score, a margin of error was permitted. Specifically, an object was classified as a “hit” in the spatial memory test if participants positioned it either at the designated location (i.e., where the rubber duck was initially placed) or within any of the eight surrounding locations marked by red stars. If the object is put to the rest 16 locations on the grid (i.e., without any mark), we would count that trial as a “miss” in the spatial memory test. If the correct location of the object is on the corner, only 3 locations around the corner would be tolerated as “hit” locations. When the correct location of the object is on the border, only 5 locations around the correct location will be included as “hit” locations.

We conducted the same models with LME toolbox as the primary results only with spatial hit, spatial error, and general spatial memory score as the dependent variables (Barr, 2013; Barr et al., 2013; Bates et al., 2015). However, these models could not pass model diagnostics. For instance, the residuals of the models do not fit a normal distribution. Therefore, we implemented the brm function of the package BRMS in R (Bürkner, 2017) to model spatial hit, spatial error, and general spatial memory score as dependent variables. We used the default priors of the BRMS package (Cauchy priors and LKJ priors for correlation parameters). The model was fit running four chains with 10000 iterations each (5000 warm-ups) and inspected for convergence. Coefficients of the effects were deemed statistically significant if the associated 95% posterior credible intervals were non-overlapping with zero.

$Spatial hit/Spatial error/General spatial memory score \sim autonomy*reward*cultural\_group+\left( 1+autonomy*reward \right| sub)$

To match the analysis with recognition memory accuracy, this same model was also conducted on the data set including all participants, high achievers and low achievers respectively.

**Results**

All results for the spatial memory test can be found in Table S1-3. The effects found significant with the models will be described in detail.

***Spatial hit***

When all participants were included, we found largely similar results for spatial memory (i.e. spatial hit) as for recognition memory (reported in the main text). Specifically, we found a significant autonomy effect, reward effect, and interaction effect between reward and cultural group on spatial hit (Table S1; Figure S2A&B). This indicated that the presence of autonomy was beneficial for spatial hit. The same was true for the presence of rewards, also when dividing each cultural group into high and low achievers (Table S2; Figure S3A-D). However, different from the recognition memory results reported in the main text, we also found a cultural group difference in spatial hit: the Chinese group had a lower spatial hit than the Dutch group. Since we also found an interaction effect between reward and cultural group on spatial hit, we conducted follow-up analyses with the *emmeans* package in R (Lenth, 2022). It was observed that the Chinese group performed worse on spatial hit than the Dutch group when they did not receive rewards (95%CI [-0.704, -0.140]), but not when they received rewards (95%CI [-0.443, 0.065]; Figure S1).

***Spatial error***

When looking at spatial error (Table S1; Figure S2C&D), we found similar results for the main effects of autonomy, reward, and cultural group as for spatial hit. Compared with the results on spatial hit, there was also no interaction between cultural group and reward on spatial error. Additionally, we also implemented the same model after dividing participants into high and low achievers based on their recognition memory accuracy (Table S3; Figure S3E-F), and similar results were yielded with the analysis including all participants.

**General spatial memory score**

When looking at the general spatial memory score, we found all 3 main effects of cultural group, autonomy, and rewards (Table S1; Figure S2E&F) when including all participants. These effects remained significant after splitting each cultural group into high and low achievers. Different from results with memory accuracy as the dependent variable, the Dutch group (63.16% ± 10.09) performed better on spatial memory tests than the Chinese group (56.55% ± 10.19).

**Conclusions**

Taken together, the results of the spatial memory test largely mirror the results of the recognition memory test reported in the main text. To be more specific, the beneficial effect of reward on memory was stronger for Chinese students than for Dutch students, whereas the influence of autonomy on memory demonstrated a uniform effect across different cultural groups. However, the results on spatial memory tests deviated from the results on recognition memory in one respect: we found Dutch students performed better than Chinese students in the spatial memory test. This group difference between Chinese and Dutch students on spatial memory performance might be explained by a finding that over-challenging tasks usually weaken learning motivation of Chinese students but not of Western students (Moneta, 2004). In our case, the spatial memory test was more difficult than the recognition memory test. This might explain why Chinese students had a worse performance than the Dutch students in the spatial memory test, but not in the recognition memory test.

**Table S1 BRMS results with Spatial memory as the dependent variable**

| Effect of interests | All participants | High achievers | Low achievers |
| --- | --- | --- | --- |
| Dependent variable: Spatial hit |  | | |
| Autonomy | **95%CI [-0.180, -0.092]** | **95%CI [-0.190, -0.075]** | **95%CI [-0.214, -0.069]** |
| Reward | **95%CI [0.210, 0.105]** | **95%CI [-0.146, -0.062]** | **95%CI [-0.312, -0.112]** |
| Cultural group | **95%CI [-0.278, -0.036]** | **95%CI [-0.286, -0.005]** | **95%CI [-0.336, -0.010]** |
| Autonomy * Reward | 95%CI [-0.051, 0.009] | 95%CI [-0.066, 0.013] | 95%CI [-0.062, 0.034] |
| Reward * Cultural group | **95%CI [-0.110, -0.005]** | 95%CI [-0.196, 0.263] | **95%CI [-0.212, -0.011]** |
| Autonomy * Cultural group | 95%CI [-0.011, 0.076] | 95%CI [-0.050, 0.033] | 95%CI [-0.057, 0.087] |
| Autonomy * Reward * Cultural group | 95%CI [-0.010, 0.050] | 95%CI [-0.022, 0.057] | 95%CI [-0.025, 0.071] |
| Dependent variable: Spatial error |  | | |
| Autonomy | **95%CI [0.023, 0.042]** | **95%CI [0.022, 0.048]** | **95%CI [0.014, 0.045]** |
| Reward | **95%CI [0.022, 0.044]** | **95%CI [0.016, 0.036]** | **95%CI [0.021, 0.061]** |
| Cultural group | **95%CI [0.009, 0.066]** | **95%CI [0.005, 0.079]** | **95%CI [0.002, 0.067]** |
| Autonomy * Reward | 95%CI [-0.002, 0.010] | 95%CI [-0.002, 0.016] | 95%CI [-0.007, 0.011] |
| Reward * Cultural group | 95%CI [-0.001, 0.021] | 95%CI [-0.008, 0.012] | 95%CI [-0.002, 0.039] |
| Autonomy * Cultural group | 95%CI [-0.018, 0.001] | 95%CI [-0.024, 0.002] | 95%CI [-0.022, 0.008] |
| Autonomy * Reward * Cultural group | 95%CI [-0.011, 0.001] | 95%CI [-0.014, 0.004] | 95%CI [-0.013, 0.004] |
| Dependent variable: General spatial test score | |  |  |
| Autonomy | **95%CI [-0.155, -0.081]** | **95%CI [-0.176, -0.089]** | **95%CI [-0.165, -0.042]** |
| Reward | **95%CI [-0.146, -0.072]** | **95%CI [-0.137, -0.052]** | **95%CI [-0.186, -0.060]** |
| Cultural group | **95%CI [-0.240, -0.051]** | **95%CI [-0.283, -0.034]** | **95%CI [-0.223, -0.049]** |
| Autonomy * Reward | 95%CI [-0.048, 0.004] | 95%CI [-0.077, 0.001] | 95%CI [-0.047, 0.028] |
| Reward * Cultural group | 95%CI [-0.071, 0.003] | 95%CI [-0.064, 0.020] | 95%CI [-0.111, 0.015] |
| Autonomy * Cultural group | 95%CI [-0.006, 0.068] | 95%CI [-0.005, 0.082] | 95%CI [-0.039, 0.086] |
| Autonomy * Reward * Cultural group | 95%CI [-0.014, 0.038] | 95%CI [-0.020, 0.058] | 95%CI [-0.032, 0.044] |

* Bold font indicates significant effects

**Table S2 Mean and standard deviation for spatial hit**

|  |  |  |  |  | **High achievers** | | | | **Low achievers** | | | |
| --- | --- | --- | --- | --- | --- | --- | --- | --- | --- | --- | --- | --- |
|  | Chinese | | Dutch | | Chinese | | Dutch | | Chinese | | Dutch | |
| *Main factors* | *M (%)* | *SD* | *M (%)* | *SD* | *M (%)* | *SD* | *M (%)* | *SD* | *M (%)* | *SD* | *M (%)* | *SD* |
| MOVE | 26.92 | 10.93 | 34.12 | 12.46 | 31.65 | 10.93 | 39.92 | 9.89 | 21.98 | 8.67 | 28.05 | 12.15 |
| FOLLOW | 23.20 | 9.48 | 27.51 | 11.74 | 28.20 | 9.08 | 32.20 | 10.88 | 17.97 | 6.77 | 22.62 | 10.77 |
| REWARD | 28.68 | 11.35 | 32.71 | 11.87 | 32.21 | 9.13 | 38.05 | 10.48 | 24.99 | 12.43 | 27.12 | 10.78 |
| NO REWARD | 21.51 | 11.07 | 29.02 | 11.50 | 27.77 | 10.77 | 34.03 | 9.12 | 14.96 | 6.88 | 23.78 | 11.56 |
| *Autonomy * Reward* | *M (%)* | *SD* | *M (%)* | *SD* | *M (%)* | *SD* | *M (%)* | *SD* | *M (%)* | *SD* | *M (%)* | *SD* |
| MOVE/ REWARD | 30.61 | 13.28 | 35.26 | 12.75 | 33.71 | 10.91 | 41.02 | 10.69 | 27.36 | 14.95 | 29.24 | 12.11 |
| MOVE/NO REWARD | 23.05 | 12.28 | 32.94 | 13.06 | 29.51 | 12.42 | 38.80 | 9.91 | 16.30 | 7.81 | 26.81 | 13.33 |
| FOLLOW/REWARD | 26.60 | 11.09 | 29.91 | 13.27 | 30.55 | 9.31 | 34.98 | 12.94 | 22.48 | 11.48 | 24.62 | 11.68 |
| FOLLOW/NO REWARD | 19.83 | 11.06 | 25.08 | 11.87 | 25.89 | 10.82 | 29.40 | 10.41 | 13.50 | 7.17 | 20.56 | 11.82 |

**Table S3 Mean and standard deviation for spatial error**

|  |  |  |  |  | **High achievers** | | | | **Low achievers** | | | |
| --- | --- | --- | --- | --- | --- | --- | --- | --- | --- | --- | --- | --- |
|  | Chinese | | Dutch | | Chinese | | Dutch | | Chinese | | Dutch | |
| *Main factors* | *M* | *SD* | *M* | *SD* | *M* | *SD* | *M* | *SD* | *M* | *SD* | *M* | *SD* |
| MOVE | 1.45 | 0.30 | 1.25 | 0.30 | 1.31 | 0.31 | 1.09 | 0.24 | 1.59 | 0.22 | 1.42 | 0.28 |
| FOLLOW | 1.54 | 0.26 | 1.41 | 0.29 | 1.41 | 0.27 | 1.27 | 0.26 | 1.67 | 0.17 | 1.56 | 0.25 |
| REWARD | 1.41 | 0.29 | 1.29 | 0.30 | 1.31 | 0.27 | 1.14 | 0.26 | 1.52 | 0.28 | 1.44 | 0.26 |
| NO REWARD | 1.57 | 0.30 | 1.38 | 0.28 | 1.42 | 0.30 | 1.23 | 0.23 | 1.74 | 0.18 | 1.53 | 0.26 |
| *Autonomy * Reward* | *M* | *SD* | *M* | *SD* | *M* | *SD* | *M* | *SD* | *M* | *SD* | *M* | *SD* |
| MOVE/EXTRA REWARD | 1.37 | 0.33 | 1.23 | 0.33 | 1.26 | 0.30 | 1.07 | 0.26 | 1.48 | 0.33 | 1.39 | 0.32 |
| MOVE/NO EXTRA REWARD | 1.53 | 0.32 | 1.28 | 0.30 | 1.36 | 0.33 | 1.12 | 0.24 | 1.70 | 0.22 | 1.44 | 0.28 |
| FOLLOW/EXTRA REWARD | 1.46 | 0.29 | 1.35 | 0.32 | 1.36 | 0.27 | 1.20 | 0.31 | 1.56 | 0.27 | 1.51 | 0.25 |
| FOLLOW/NO EXTRA REWARD | 1.62 | 0.30 | 1.48 | 0.30 | 1.47 | 0.31 | 1.34 | 0.27 | 1.78 | 0.20 | 1.61 | 0.29 |

Note: The mean represents the Euclidean distance calculated between the response location and the correct location of each object in the spatial memory test.

**Table S4 Mean and standard deviation for general spatial score**

|  |  |  |  |  | **High Achievers** | | | | **Low Achievers** | | | |
| --- | --- | --- | --- | --- | --- | --- | --- | --- | --- | --- | --- | --- |
|  | Chinese | | Dutch | | Chinese | | Dutch | | Chinese | | Dutch | |
| *Main factors* | *M* | *SD* | *M* | *SD* | *M* | *SD* | *M* | *SD* | *M* | *SD* | *M* | *SD* |
| MOVE | 58.52% | 11.49% | 66.36% | 10.86% | 64.81% | 10.63% | 72.98% | 8.09% | 51.95% | 8.34% | 59.44% | 8.95% |
| FOLLOW | 54.44% | 10.15% | 59.83% | 10.97% | 60.60% | 9.35% | 65.98% | 9.40% | 48.00% | 6.26% | 53.40% | 8.65% |
| REWARD | 59.84% | 10.50% | 64.82% | 11.14% | 65.36% | 9.25% | 71.03% | 9.38% | 54.07% | 8.53% | 58.32% | 9.02% |
| NO REWARD | 53.16% | 11.80% | 61.49% | 10.28% | 60.14% | 10.77% | 67.92% | 8.00% | 45.86% | 7.82% | 54.77% | 7.85% |
| *Autonomy * Reward* | *M* | *SD* | *M* | *SD* | *M* | *SD* | *M* | *SD* | *M* | *SD* | *M* | *SD* |
| MOVE/EXTRA REWARD | 61.56% | 11.71% | 67.17% | 12.12% | 67.03% | 9.97% | 73.29% | 9.61% | 55.84% | 10.77% | 60.77% | 11.28% |
| MOVE/NO EXTRA REWARD | 55.38% | 13.69% | 65.50% | 10.71% | 62.56% | 12.76% | 72.62% | 7.60% | 47.87% | 10.31% | 58.05% | 8.13% |
| FOLLOW/EXTRA REWARD | 58.14% | 11.06% | 62.19% | 12.34% | 63.69% | 10.12% | 68.62% | 11.33% | 52.33% | 8.92% | 55.47% | 9.59% |
| FOLLOW/NO EXTRA REWARD | 50.81% | 12.00% | 57.43% | 11.59% | 57.54% | 10.61% | 63.33% | 9.58% | 43.78% | 9.07% | 51.27% | 10.35% |


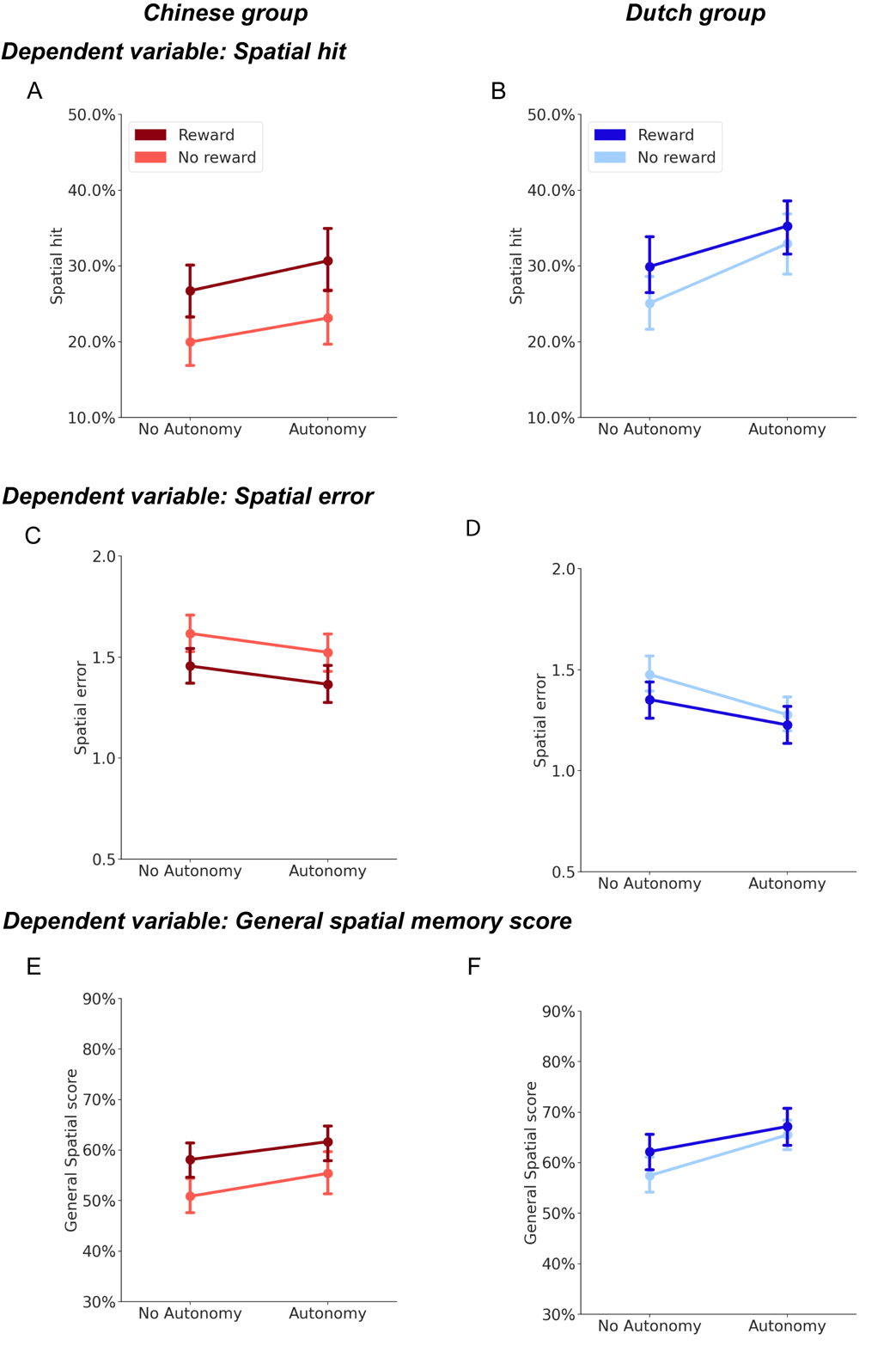


**Figure S2 Results on spatial hits and spatial error (Table S1)**

**A.** For the Chinese group, the spatial hit is plotted as a function of autonomy and reward. The red color represents the Chinese cultural group. The dark red color represents the reward condition, while the light red color represents the no reward condition. The error bars represent the standard error of the means (*SEM*). **B.** As in A, the spatial hit is plotted as a function of autonomy and reward for the Dutch group. The blue color represents the Dutch cultural group. The dark blue color represents the reward condition, while the light blue color represents the no reward condition. Other conventions are the same as in Figure S1A. **C.** For Chinese students, spatial error is plotted as a function of autonomy and reward. It should be noted that a lower spatial error reflects better spatial memory performance. All conventions are the same as in Figure S1A. **D.** For the Dutch group, spatial error is plotted the same as in Figure S1B. **E & F.** The conventions are the same as the panel of A & B, only with the y-axis representing general spatial memory score instead of spatial hit.


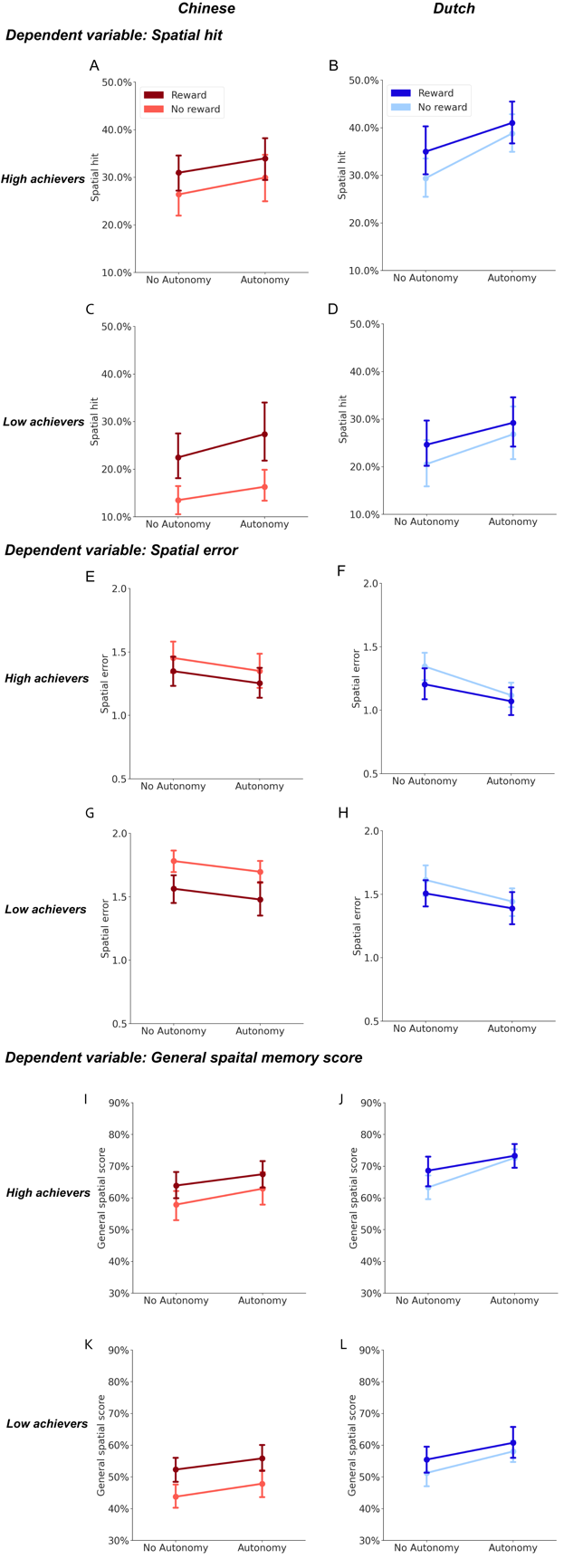


**Figure S3 The results respectively on high and low achievers are listed in Table S3.**

All conventions are the same as in Figure S1.

**A.** For the Chinese high achievers, the spatial hit is plotted as a function of autonomy and reward. **B.** For the Dutch high achievers, the spatial hit is plotted as a function of autonomy and reward **C.** For Chinese low achievers, the spatial hit is plotted the same as in Figure S2A. **D.** For the Dutch low achievers, the spatial hit is plotted the same as in Figure S2B. **E-H.** The conventions are the same as the panel of A & B & C & D, only with the y-axis representing spatial error instead of spatial hit. **I-J.** The conventions are the same as the panel of A & B & C & D, only with the y-axis representing general spatial memory score instead of spatial hit.

**References**

Barr, D. J. (2013). Random effects structure for testing interactions in linear mixed-effects models. *Front Psychol*, *4*, 328. https://doi.org/10.3389/fpsyg.2013.00328

Barr, D. J., Levy, R., Scheepers, C., & Tily, H. J. (2013). Random effects structure for confirmatory hypothesis testing: Keep it maximal. *J Mem Lang*, *68*(3). https://doi.org/10.1016/j.jml.2012.11.001

Bates, D., Mächler, M., Bolker, B., & Walker, S. (2015). Fitting Linear Mixed-Effects Models Using lme4. *Journal of Statistical Software*, *67*(1). https://doi.org/10.18637/jss.v067.i01

Bürkner, P.-C. (2017). brms: An R Package for Bayesian Multilevel Models Using Stan. *Journal of Statistical Software*, *80*(1). https://doi.org/10.18637/jss.v080.i01

Lenth, Russell V. (2022). emmeans: Estimated Marginal Means, aka Least-Squares Means. *R package version 1.8.3*. <https://CRAN.R-project.org/package=emmeans>

Moneta, G. B. (2004). The flow model of intrinsic motivation in Chinese: Cultural and personal moderators. *Journal of Happiness Studies*(5), 181-217.
