# Supplementary material for: A cross-cultural comparison of intrinsic and extrinsic motivational drives for learning
Source: Cogn Affect Behav Neurosci. 2024 Oct 18;25(1):25–44. doi: 10.3758/s13415-024-01228-2 (PMC11805854; doi:10.3758/s13415-024-01228-2)
Supplement: Supplementary file 8 — (DOCX 17 kb) [file 13415_2024_1228_MOESM5_ESM.docx]

**Supplementary Material 2: signal detection theory based analysis**

**Methods**

Meanwhile, there might be cross-cultural differences in response biases (Leger & Gutchess, 2021). This difference in response bias might contribute to the differences in reward effect on memory accuracy between cultural groups. To rule out this possibility, we employed signal detection theory to calculate both *d’* and *C* parameters, as detailed by Hautus et al. (2021; https://camel.psyc.vt.edu/models/recognition/index.shtml). In the current experiment, both the manipulation of autonomy (MOVE/FOLLOW) and rewards (REWARD/NO REWARD) were within-subject. Only the comparison of cultural groups was between-subject. In the learning phase of the experiment, each participant learned a total of 300 objects, divided into 75 objects per experimental condition. During the memory test, these objects were presented in random order, intermixed with 300 filler objects (foils). Since these foil items do not belong to any of the four conditions, we could only calculate *d’* and *C* at for overall performance at a participant level, but not per condition.

In order to compute *d’* and *C*, we first calculated the hit rate and false alarm for each participant.

$$Hit rate={Hit object number}_{Old}/{Total object number}_{Old}$$

$$False alarm={Hit object number}_{New}/{Total object number}_{New}$$

We implemented *z* transformation for Hit rate (*Z_H_*) and False alarm (*Z_FA_*) for each participant. Then, *d’* and *C* for each participant were computed as follows.

$$d^{'}= Z_{H}-Z_{FA}$$

$C= -1/2[Z_{H}+Z_{FA}$]

According to signal detection theory, d’ represents the discriminability/sensitivity. The higher the *d’* is for a participant, the better the participant is at discriminating between old and new objects. *C* represents the bias. If *C* is higher than 0, this suggests that participants had a conservative bias, meaning that participants tended to guess objects were NEW in the memory test. If *C* is lower than 0, this indicates that participants had a liberal bias, meaning that participants tended to guess objects were OLD in the memory test. With *d’* and *C* as dependent variables, we respectively conducted independent sample t-tests between Dutch and Chinese groups.

**Results**

In the results of *t*-tests, we found that the Dutch group (1.96 + 0.50) showed a higher *d’*(discriminability) than the Chinese group (1.51 + 0.58), *t* (86.40) = 3.90, *p* < 0.001. This suggests that Dutch students were better at discriminating between OLD and NEW objects than Chinese students. We also did the same analysis for *C* (bias) and found no difference between cultural groups (Dutch group: 0.40 + 0.30; Chinese group: 0.34 + 0.43.), *t* (78.67) = -0.79, *p* = 0.43. This suggested that there were no group differences in response bias, validating that our findings regarding the different effects of reward on memory accuracy between cultural groups could not be explained by bias alone.

The results partially aligned with previous work Leger and Gutchess (2021), in which they found that participants from North America were better at discriminating between OLD and NEW objects (*d’*) than participants from East Asia. They also found that participants from North America had a higher bias to respond to an object as OLD than East Asian participants. Besides, in our study, we did not find differences in response bias (*C*) between cultural groups. To summarize, the cross-cultural differences in the reward effect might not be caused by differences in response bias between cultural groups. The current study builds upon the findings of Leger & Gutchess (2021), demonstrating that cultural differences extend beyond memory specificity also to include the effects of intrinsic and extrinsic motivation on memory.

**References**

Hautus, M. J., Macmillan, N. A., & Creelman, C. D. (2021). *Detection theory: A user's guide*. Routledge.

Leger, K. R., & Gutchess, A. (2021). Cross-Cultural Differences in Memory Specificity: Investigation of Candidate Mechanisms. *J Appl Res Mem Cogn*, *10*(1), 33-43. https://doi.org/10.1016/j.jarmac.2020.08.016
