# Supplementary material for: A cross-cultural comparison of intrinsic and extrinsic motivational drives for learning
Source: Cogn Affect Behav Neurosci. 2024 Oct 18;25(1):25–44. doi: 10.3758/s13415-024-01228-2 (PMC11805854; doi:10.3758/s13415-024-01228-2)
Supplement: Supplementary file 9 — (DOCX 20 kb) [file 13415_2024_1228_MOESM6_ESM.docx]

**Supplementary Material 3: Analysis of Recognition Memory Confidence**

**Methods**

In the main manuscript, we labeled responses 1 and 2 were labeled as 'New,' and responses 3 and 4 were labeled as 'Old.' This binary classification was chosen to simplify the statistical analysis and to focus on the primary (preregistered) research question of whether participants could accurately distinguish between new and old items. In other words: it allows for a more straightforward interpretation of the recognition memory performance.

In this supplementary analysis, we implemented the same model as the main analysis on recognition memory accuracy on the Likert scale for memory confidence. We labeled “Definitely Old” -> 4, “Probably Old” -> 3, “Probably New” -> 2, “Definitely New” -> 1. Instead of using the glmer toolbox in R, we used the clmm toolbox (<https://search.r-project.org/CRAN/refmans/ordinal/html/clmm.html>) since the dependent variable was ordinal. In this way, the higher the Likert scale is, the more confident the participants are in remembering the objects. We did not report this in the main manuscript, since the results were very similar to the results using the binary format, but the results can be found in Supplementary Material 3.

**Results**

We also found the main effects of autonomy and reward and the interaction between the factor of cultural groups and rewards on the Likert scale. We did not report this in the manuscript since the results highly mirrored the results on memory accuracy. The results from this model are reported in tables here. These codes are also available in the open access codes for future researchers to check.

**Table S5 CLMM results with the Likert scale for recognition memory as the dependent variable**

| Effect of interests | *β* | *z* | *p* |
| --- | --- | --- | --- |
| Autonomy | -0.23 | -7.33 | <0.001*** |
| Reward | -0.17 | -5.68 | <0.001*** |
| Cultural group | -0.12 | -1.62 | 0.11 |
| Autonomy * Reward | -0.02 | -1.48 | 0.19 |
| Reward * Cultural group | -0.08 | -2.56 | 0.01* |
| Autonomy * Cultural group | 0.04 | 1.25 | 0.21 |
| Autonomy * Reward * Cultural group | 0.02 | 1.29 | 0.14 |

**Table S6 Mean and standard deviation for the Likert scale for recognition memory**

|  | Chinese | | Dutch | |
| --- | --- | --- | --- | --- |
| *Main factors* | *M* | *SD* | *M* | *SD* |
| MOVE | 3.07 | 0.40 | 3.27 | 0.36 |
| FOLLOW | 2.85 | 0.44 | 2.98 | 0.41 |
| REWARD | 3.11 | 0.36 | 3.18 | 0.34 |
| NO REWARD | 2.82 | 0.52 | 3.08 | 0.37 |
| *Autonomy * Reward* | *M* | *SD* | *M* | *SD* |
| MOVE/EXTRA REWARD | 3.21 | 0.38 | 3.29 | 0.37 |
| MOVE/NO EXTRA REWARD | 2.93 | 0.53 | 3.24 | 0.38 |
| FOLLOW/EXTRA REWARD | 3.00 | 0.41 | 3.06 | 0.43 |
| FOLLOW/NO EXTRA REWARD | 2.71 | 0.56 | 2.91 | 0.44 |

Notes: The dependent variable for this model is the Likert scale (e.g., “Definitely Old” -> 4, “Probably Old” -> 3, “Probably New” -> 2, “Definitely New” -> 1). The higher the Likert scale is, the more confident participants are that the objects were seen in the learning task.

To follow up, we also compared the Likert scale for the REWARD and NO REWARD conditions respectively for the Dutch group and the Chinese group (Figure 3A) with the *emmeans* package in R (Lenth, 2022). It was found that the facilitatory effect of reward (REWARD – NO REWARD) on Likert scale was significant for both the Chinese group (*β* = 0.48, *z* = 5.82, *p* < 0.001) and the Dutch group (*β* = 0.19, *z* = 2.23, *p* = 0.03). This reward effect on Likert scale was stronger for the Chinese group compared with the Dutch group. Alternatively, we also compared Likert scale between the Chinese and Dutch groups under both REWARD and NO REWARD conditions respectively (Figure 2). It was found that under reward conditions, Likert scales were at a similar level between the Dutch and Chinese groups (*β* = 0.09, *z* = 0.61, *p* = 0.54). However, under the NO REWARD condition, the Dutch group performed with a higher confidence than the Chinese group (*β* = 0.39, *z* = 2.20, *p* = 0.03) in the recognition memory test.

In summary, the results of the CLMM model with Likert scale of recognition memory as the dependent variable mirrored the results from the main manuscript, where we used the binary dependent variable, recognition memory accuracy, as the dependent variable.
